# A transcriptome-based global map of signaling pathways in the ovarian cancer microenvironment associated with clinical outcome

**DOI:** 10.1186/s13059-016-0956-6

**Published:** 2016-05-23

**Authors:** Silke Reinartz, Florian Finkernagel, Till Adhikary, Verena Rohnalter, Tim Schumann, Yvonne Schober, W. Andreas Nockher, Andrea Nist, Thorsten Stiewe, Julia M. Jansen, Uwe Wagner, Sabine Müller-Brüsselbach, Rolf Müller

**Affiliations:** Clinic for Gynecology, Gynecological Oncology and Gynecological Endocrinology, Center for Tumor Biology and Immunology (ZTI), Philipps University, Marburg, Germany; Institute of Molecular Biology and Tumor Research (IMT), Center for Tumor Biology and Immunology (ZTI), Philipps University, Hans-Meerwein-Str. 3, Marburg, 35043 Germany; Metabolomics Core Facility and Institute of Laboratory Medicine and Pathobiochemistry, Center for Tumor Biology and Immunology (ZTI), Philipps University, Marburg, Germany; Genomics Core Facility, Center for Tumor Biology and Immunology (ZTI), Philipps University, Marburg, Germany

**Keywords:** Ovarian carcinoma, Tumor-associated macrophages, Tumor microenvironment, Malignancy-associated ascites, Signaling network, Arachidonic acid, IL-10, TGFβ

## Abstract

**Background:**

Soluble protein and lipid mediators play essential roles in the tumor environment, but their cellular origins, targets, and clinical relevance are only partially known. We have addressed this question for the most abundant cell types in human ovarian carcinoma ascites, namely tumor cells and tumor-associated macrophages.

**Results:**

Transcriptome-derived datasets were adjusted for errors caused by contaminating cell types by an algorithm using expression data derived from pure cell types as references. These data were utilized to construct a network of autocrine and paracrine signaling pathways comprising 358 common and 58 patient-specific signaling mediators and their receptors. RNA sequencing based predictions were confirmed for several proteins and lipid mediators. Published expression microarray results for 1018 patients were used to establish clinical correlations for a number of components with distinct cellular origins and target cells. Clear associations with early relapse were found for STAT3-inducing cytokines, specific components of WNT and fibroblast growth factor signaling, ephrin and semaphorin axon guidance molecules, and TGFβ/BMP-triggered pathways. An association with early relapse was also observed for secretory macrophage-derived phospholipase PLA_2_G_7_, its product arachidonic acid (AA) and signaling pathways controlled by the AA metabolites PGE_2_, PGI_2_, and LTB_4_. By contrast, the genes encoding norrin and its receptor frizzled 4, both selectively expressed by cancer cells and previously not linked to tumor suppression, show a striking association with a favorable clinical course.

**Conclusions:**

We have established a signaling network operating in the ovarian cancer microenvironment with previously unidentified pathways and have defined clinically relevant components within this network.

**Electronic supplementary material:**

The online version of this article (doi:10.1186/s13059-016-0956-6) contains supplementary material, which is available to authorized users.

## Background

Ovarian carcinoma ranks fifth as the cause of death from cancer in women with >40,000 new cases annually in the European Union [1]. Ovarian cancer has a dire prognosis with an overall five-year survival rate of <25 %. The World Health Organization classification distinguishes six major entities of ovarian tumor [1]. Of these, high grade serous ovarian carcinoma is not only the most common ovarian cancer, but also the deadliest of all gynecological malignancies. Up to 95 % of these patients with advanced stage disease present with tumor masses in the abdomen beyond the pelvis and/or lymph node metastases (FIGO stage III) or organs outside the peritoneal cavity (stage IV). These facts clearly attest to the malicious nature of this disease and identify serous ovarian cancer as a major health issue world-wide.

Several features contribute to the fatal nature of serous ovarian carcinoma, some of which make this cancer unique among all human tumors [2]. Tumor cells are often shed at a very early stage of the disease. Even at a stage when primary tumors are still confined to one or both of the ovaries, cancer cells can be detected in peritoneal lavage fluid (stage IC). While blood and the lymphatic system are major routes of dissemenation in other cancers, the spread of ovarian tumor cells is driven by the peritoneal fluid. Ovarian cancer cells then adhere to and superficially invade the omentum and the serous membranes lining other peritoneal organs, giving rise to tumor foci growing into the open space of the peritoneal cavity [2]. The peritoneal microenvironment, which is formed by the ascites building up in the peritoneal cavity, is an essential determinant of metastatic disease progression due to its tumor-promoting soluble factors [3], exosomes [4], highly tumorigenic cancer cells [5], and different types of immune cells, including pro-tumorigenic tumor-associated macrophages (TAMs) [6, 7].

TAMs are blood monocyte-derived cells polarized by factors of the tumor microenvironment to adopt phenotypes that clearly deviate from classically or alternatively activated macrophages [8–10]. This also applies to TAMs isolated from ovarian cancer ascites [7]. TAMs are pro-tumorigenic and promote all aspects of cancer growth and progression, including tumor cell proliferation, invasion, angiogenesis, formation of metastasis, and immune suppression [8, 9, 11, 12]. The critical role of TAMs has been demonstrated in numerous mouse models and is strongly supported by the correlation of clinical outcome with intratumoral macrophage density in different types of cancer [11], including ovarian carcinoma [13]. Consistent with these observations, the presence of CD163^high^ TAMs in the malignancy-associated ascites showed a strong correlation with early relapse of serous ovarian carcinoma after first-line therapy [7].

Cytokines and growth factors released into the tumor microenvironment are pivotal to all aspects of tumor progression. Tumor growth, cancer dissemination, and immune escape are promoted by a plethora of growth factors and cytokines that are also found in ovarian cancer ascites [7, 14–16]. These factors (1) induce cell proliferation, such as epidermal growth factor (EGF) family members and interleukin (IL)-6, (2) trigger angiogenesis, e.g. vascular EGF (VEGF), basic FGF, and IL-8, (3) attract immune cells to the tumor, in particular chemokines of the CCL and CXCL families [17], and (4) polarize these to pro-tumorigenic and immune suppressive cells, for example VEGF, IL-6, IL-10, and LIF [18]. One of the central factors promoting tumor progression is transforming growth factor (TGF) β [19], which triggers epithelial-mesenchymal transition (EMT), cancer cell invasion, metastasis, and immune suppression. Soluble factors may also play a role in promoting stemness properties, for example, KIT ligand and R-spondins as ligands for CD117 [20] and LGR5 [21, 22], respectively. Several growth factors and cytokines also inhibit apoptosis and the efficacy of chemotherapeutic drugs, such as IL-6, IL-10, and TGFβ [23]. Finally, ascites fluid promotes its own accumulation, mainly though the action of VEGF as a vascular permeability factor [24].

A recent study evaluating publicly available genomic data has identified a number of clinical associations of signaling loops established by polypeptide ligands and their receptors in advanced ovarian cancer, including TGFβ, PDGF, VEGF, ephrin, CXCL12, and CCL chemokines [25]. However, since all expression data were derived from solid tumor tissue, tumor and host cell-specific contributions could not be analyzed, which also suggests that pathways involving host cells as major constituent were missed.

Molecules generated by the cleavage of phospholipids and present in malignant effusions represent another important class of soluble cancer-promoting mediators, in particular lysophosphatitic acid (LPA) [26–31] and arachidonic acid (AA)-derived eicosanoids [32–34]. The latter include prostanoids, hydroxyeicosatetraenoic acids (HETEs), and leukotrienes that are produced from AA by enzymatic cascades initiated either by cyclooxygenases or lipoxygenases. The importance of lipid mediators for tumorigenesis is exemplified by LPA as a mediator of cancer cell invasion and chemoresistance [28, 31, 35] and prostaglandin E_2_ as an immune suppressor and trigger of angiogenesis [36].

To be able to understand the biological role of the large number of soluble mediators in the tumor microenvironment, a global picture of their cellular origins and targets is indispensible, but currently not available. One possibility is to address this question by a genomic approach. However, although transcriptomic data for a large number of solid tumor samples from ovarian cancer patients have been published [37–39], these are not suitable to determine expression levels in tumor cells and specific tumor-associated host cells. We have addressed this issue by determining the transcriptomes for the major cell types of serous ovarian carcinoma, i.e. tumor cells and TAMs, purified from the ascites of patients. Ascites-associated cancer cells occur as single cells or multicellular spheroids and are likely to be responsible for peritoneal dissemination and to contribute to relapse of the disease [2]. In spite of their clinical relevance, genome-wide studies have not been performed with ascites-associated cells from ovarian cancer.

In the present study, we determined the transcriptome for tumor cells and TAMs from ovarian cancer ascites and used these data to construct a network comprising cytokines, growth factors, lipid mediators, and their receptors, which we confirmed for several components at the level of the respective proteins or lipids. These data defined a multitude of specific signaling pathways between tumor cells and TAMs as well as cell-type restricted, autocrine mechanisms. Furthermore, by establishing correlations with disease progression, we provide clear evidence for the biological relevance of soluble mediators in the ovarian cancer microenvironment. Thus, our data identified a highly significant link to disease recurrence not only for several cytokines and AA, but also a striking synergistic association between these proteins and AA. These findings underscore the biological relevance of functional interactions in the ovarian cancer microenvironment.

## Results

### Characterization of patient samples

Tumor cells and/or TAMs were isolated from the ascites of 28 patients with high grade serous ovarian carcinoma and one patient with serous borderline tumor (low grade carcinoma) (Additional file 4: Table S1). If feasible, tumor cell spheroids from the same patients were fractionated according to size (single cells: “sc”; small: <30 μm, “s”; medium: 30–40 μm, “m”; large: >40 μm, “L”). Surprisingly, small and large spheroids from the same patients frequently showed clear genetic and biological differences (Additional file 4: Table S2). For instance, small spheroids usually comprised pseudo-diploid cells, rapidly adhered to culture dishes in the presence of autologous ascites and were chemosensitive, whereas large spheroids were largely aneuploid, persisted as floating spheres in culture and were completely chemoresistant. Therefore, both small and large spheroids were included in all subsequent studies and analyzed separately.

### Adjustment of RNA sequencing data for contaminating cell types

A central goal of the present study was an RNA sequencing (RNA-Seq) based comparison of the expression of signaling components of tumor cells and TAMs. We focused our study on primary, non-cultured cells in order to obtain a faithful picture of the signaling network operating in vivo. However, the presence of variable amounts (0–50 %) of TAMs in isolated tumor cell fractions and vice versa may lead to incorrect conclusions in particular for genes that show a differential, cell type-specific expression. The impact of such “contaminations” on gene expression profiles is a well-known problem and has consequently been addressed by numerous published algorithms [40–50]. However, none of these fulfills all the criteria required by our specific conditions, as explained in detail in Additional file 1.

A particularly relevant aspect in this context is the mixed-polarization phenotype of ovarian cancer ascites-associated TAMs, which share only small subsets of upregulated genes with M1 and M2 macrophages (Additional file 2: Figure S1). This precludes the use of literature data obtained with canonically activated macrophages as, for example, in CIBERSORT [48]. Likewise, the transcriptome of tumor cells from ovarian cancer ascites has not been determined yet. Therefore, appropriate reference data for ascites-derived tumor cells and TAMs were not available prior the present study. Finally, most published algorithms generate estimates of the fraction of contaminating cell types, but do not adjust the TPM values in RNA-Seq datasets.

**Fig. 1:**
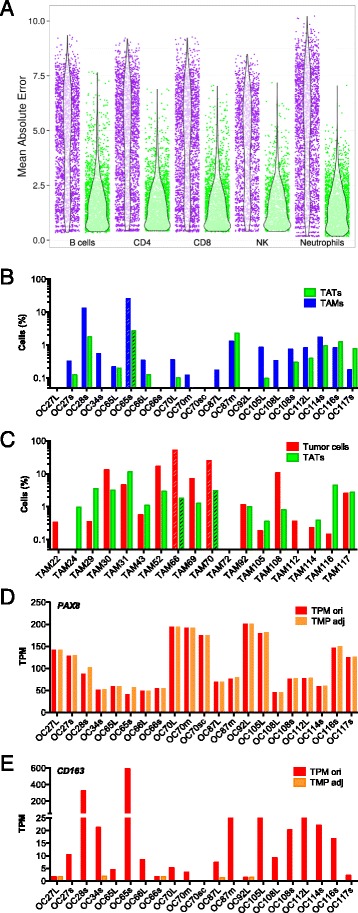
Adjustment RNA-Seq data based on RNA-Seq mixture modeling. **a** Simulation results from *in-silico* mixture of different purified immune cells with purified monocytes from dataset GSE60424 [51]. Deviation of TPM values from ground truth (unmixed sample) was quantified as the mean absolute error (MAE). *Purple*: uncorrected samples; *green*: corrected samples. Each *dot* represents one simulation with a random mixture percentage between 0 % and 50 %. *Violin plots* show the distribution of MAE values. See “Results” for description of dataset used. The algorithm was applied for estimation of contamination and data adjustment as described in Additional file 1. **b** Estimated TAM contamination of tumor samples used in the present study, based on RNA-Seq mixture modeling. **c** Estimated tumor cell contamination of TAM samples. *Striped bars* in (**b**) and (**c**) denote samples excluded from further analysis. **d**, **e** Effect of adjustment by RNA-Seq mixture modeling on marker gene expression (*PAX8*, *CD163*) in tumor cell samples. ori, original TPM values; adj, adjusted TPM

To establish a bioinformatic tool to adjust our datasets, we used a simple but highly effective approach. First, pure reference samples representing the cell type of interest (“target”) and the contaminating cell type are selected, the purity of which was confirmed by flow cytometry or other methods. RNA-Seq data for these references samples are then used to select a set of contamination marker genes, suitable for estimating the extent of contamination. Finally, the target dataset is adjusted by a linear model. A detailed description of our algorithms is found in Additional file 1. For testing our method we simulated mixtures from published RNA-Seq datasets, which showed a clear improvement, as exemplified in Fig. 1a for mixtures of purified immune cells (RNA-Seq data from GSE60424 [51]) or different tissues (Additional file 1). Furthermore, none of the previously described algorithms matched this performance (Additional file 1).

The algorithm was then applied to our set of RNA-Seq samples of tumor cells (n = 21), TAMs (n = 18), and tumor-associated T cells (TATs; n = 5). The detected contamination of tumor cell or TAM samples ranged from 0 % to 17 % (Fig. 1b, c) and was in agreement with prior analyses (as in Additional file 4: Table S2). To test the power of the algorithm, we also included RNA-Seq data from a heavily contaminated tumor sample (OC65s: 25.7 % TAMs; striped bars in Fig. 1b) and two heavily contaminated TAM samples (TAM66s: 49.4 % tumor cells and TAM70: 24.9 %; striped bars in Fig. 1c). These three samples were excluded from all subsequent experiments.

These data were used to adjust the RNA-Seq data for cross-contaminating tumor cells, TAMs, and TATs. Adjustment was successful, as exemplified in Fig. 1d and e for tumor cells. While the macrophage marker gene *CD163* was reduced, the epithelial cell marker gene *PAX8* was not. The observed increase in *PAX8* is due to the fact that TPM values represent a relative measure, thus resulting in a redistribution from reduced to non-reduced genes.

These adjusted RNA-Seq data for 20 tumor cell and 16 TAM samples (Additional file 3: Dataset S1) were analyzed for expression of two classes of mediators and their receptors: (1) cytokines and polypeptide growth factors, collectively referred to as protein mediators in the following; and (2) phospholipid breakdown products and eicosanoids functioning as lipid mediators, as described in detail below.

### Common expression of protein mediators and their receptors by tumor cells and TAMs

**Fig. 2:**
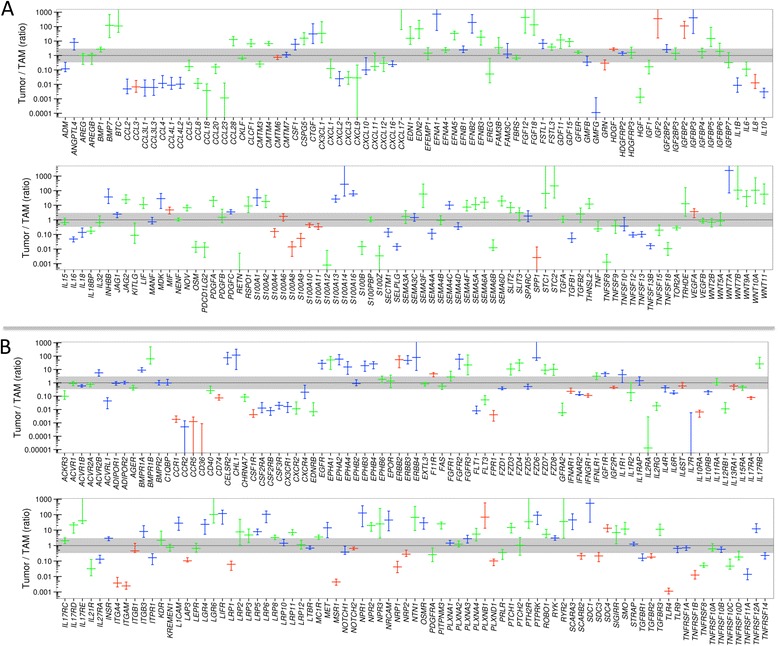
Genes coding for components of cytokine and growth factor signaling expressed in ovarian cancer cells and/or TAMs (RNA-Seq). **a** Genes coding for cytokines and growth factors. Values represent the ratio of expression in tumor cells versus TAMs (median and 95 % CI). The color code indicates the level of expression: *green*, low expression (TPM 3–20); *blue*, moderate expression (TPM 20–100); *red*, high expression (TPM >100). **b** Genes coding for cytokine/growth factor receptors. For further details see Additional file 3: Datasets S2–S5

We first established datasets of 791 genes encoding protein mediators and their receptors based on literature and database-derived data, in total 502 cytokine and growth factor genes (Additional file 3: Dataset S2) and 289 receptor genes (Additional file 3: Dataset S4). Genes with TPM values ≥3 in at least 65 % of all tumor cell or TAM samples were considered expressed and part of a common signaling network. Using these criteria, we identified 159 cytokine and 173 receptor genes to be expressed in tumor cells and/or TAMs (Fig. 2a, b; Additional file 3: Dataset S4 and S5). Genes were defined as cell type-selective if expression levels between tumor cells and TAMs differed at least threefold (thresholds indicated by the shaded areas in Fig. 2) and the individual TPM values determined for one cell type were either larger or smaller than the values for the other cell type, allowing maximum one outlier (Additional file 3: Datasets S4, S5: column “no overlap”). These datasets were further split into groups showing low (green bars in Fig. 2a, b), median (blue), or high (red) expression levels according to the observed TPM values.

**Fig. 3:**
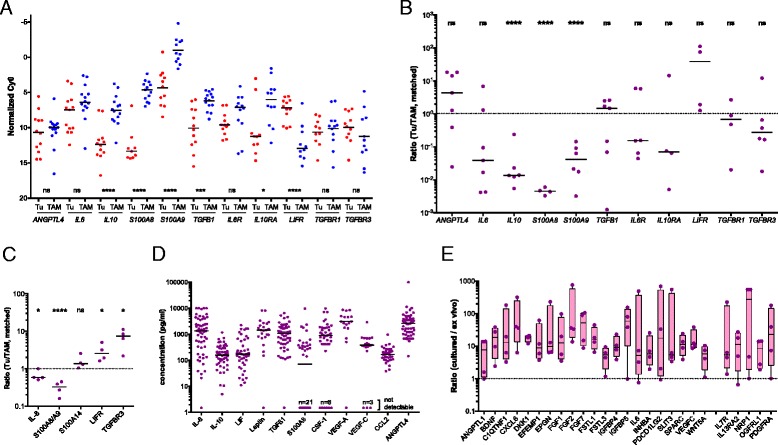
Expression of cytokines, growth factors, and their receptors in ovarian cancer ascites. **a** Validation of RNA-Seq data by RT-qPCR of tumor cell and TAM samples (each *dot* represents a different sample). **b** RT-qPCR analysis of matched tumor cell and TAM samples from the same patients (each *dot* represents a matched pair). Data are represented as the ratio of expression in tumor cells and TAMs. The *dotted line* indicates a ratio of 1. **c** FACS analysis of cytokine (intracellular IL-8, S100A8/A9, and S100A14) and receptor (LIFR and TGFBR3) expression by tumor cells and TAMs. Data in (**a**)–(**c**) were statistically analyzed by Student’s *t*-test (two-sided, unequal variance). *Horizontal bars* indicate the median. Gene names are explained in Additional file 3: Datasets S4 and S5. **d** Concentrations of cytokines and growth factors in the ascites fluid from ovarian cancer patients. Each *dot* represent a different patient, *horizontal lines* indicate the median. **e** RNA-Seq analysis of tumor cell spheroids before and after a 6-day culture in serum-free medium supplemented with 10 % autologous ascites (n = 4). The *figure* shows the ratio of matched pairs for all protein mediator-encoding genes induced under these conditions at least tenfold (each *dot* represents a matched pair; median: *horizontal bar*; 95 % CI: *box*; min–max: *whiskers*)

Differences of more than 1000-fold were observed with respect to the expression levels of different genes as well as the cell type selectivity of individual genes. These results were confirmed by RT-qPCR using a larger number of patient-derived samples for all instances tested, including a statistically highly significant preferential expression of *IL10*, *TGFB1*, *S100A8*, *S100A9*, and *IL10RA* by TAMs and *LIFR* by tumor cells (Fig. 3a). The analysis of matched tumor cell and TAM samples from the same patients are in agreement with these conclusions with the exception of *TGFB1* (Fig. 3b).

We next determined the levels of protein expression for several examples by flow cytometry of non-separated ascites samples and confirmed the preferential expression of S100A8/A9 and IL-8 in TAMs, and of LIFR and TGFBR3 in tumor cells (Fig. 3c and Additional file 2: Figure S2). Finally, we measured the levels of a number of protein mediators in the ascites of up to 40 serous ovarian cancer patients (Additional file 4: Table S3) and found readily detectable levels for all mediators shown in Fig. 3d, whereas IL4, IL12, IL13, and GM-CSF were not detectable, consistent with the RNA-Seq and RT-qPCR data (Fig. 2a and 3a). However, in a few cases, ascites levels were unexpectedly high in view of the low expression of the corresponding mRNAs in tumor cells and TAMs, e.g. IL-6 and VEGF-C (Fig. 2; Additional file 3: Datasets S3 and S5). We therefore investigated whether this apparent discrepancy could be due to differences in expression levels in unattached tumor cells in suspension, as in spheroids, and in attached tumor cells. To address this question, we performed RNA-Seq analyses for four matched pairs of uncultured and cultured spheroids. The latter were kept in serum-free medium supplemented with autologous ascites for 6 days, under which conditions the cells partly adhere to the plastic surface. The results clearly show that a small number of cytokine genes were indeed induced under these conditions, including *IL6* and *VEGFC* (Fig. 3e), while other ones, such as *IL10* and *LIF* were not. It is therefore possible that adherent tumor cells and solid tumor masses rather than floating cells are the major source of some of the ascites-associated protein mediators.

### Delineation of a common signaling network of protein mediators established by tumor cells and TAMs

**Fig. 4:**
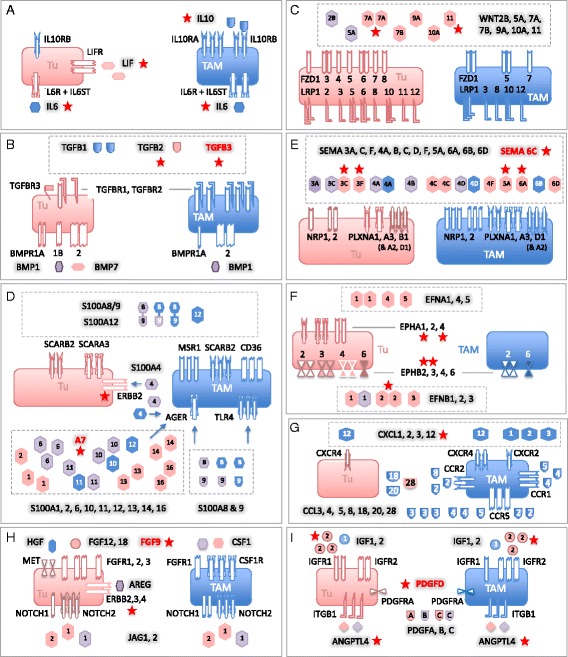
A common cytokine signaling network of ovarian cancer cells and TAMs. Ligands are represented as “free floating” symbols, receptors as membrane-associated symbols. Ligands derived from tumor cells are shown in *red*, ligands originating from TAMs in *blue*, ligands expressed by both cell types at similar levels (less than tenfold difference in TPM) in *purple*. Each ligand or receptor is represented by one or more identical symbols according to their expression levels (1, 2, and 3 symbols corresponding to *green*, *blue*, and *red*, respectively in Fig. 2). The model is based on the data in Figs. 2, 9c and Table 1 and assumes that protein levels follow gene expression. Gene names are explained in Additional file 3: Datasets S4 and S5. *Red asterisks* denote components associated with a poor clinical outcome (based on Figs. 7–9). Ligands shown in *red letters* are expressed only in a subset of patients (Table 1) and associated with a short relapse-free survival (RFS) (Fig. 9c)

Based on these data, we derived a model of a signaling network involving ovarian cancer cells and TAMs (Fig. 4). The predicted cellular origins and targets of cytokines and growth factors are also summarized in Additional file 2: Figure S3. In the following sections, we will describe the most prominent signaling pathways identified by our analyses.

(i) The STAT3-inducing cytokines IL-10, IL-6, and LIF were identified as part of the signaling network established in the present study (Fig. 4a). *IL10* and the gene encoding its receptor *IL10R* were expressed mainly by TAMs, *LIF* and *LIFR* by tumor cells, *IL6* and the genes for IL6 receptor subunits *IL6R* and *IL6ST* by both cell types.
(ii) *TGFB1*, mainly expressed by TAMs, codes for the major ligands of the TGFβ network, which also comprises tumor cell-derived *TGFB2* and *BMP7* (encoding bone morphogenetic protein 7) as well as *BMP1* expressed by both cell types (Fig. 4b). These ligands target both cell types, as suggested by the expression patterns of the *TGFBR* and *BMPR2* genes.
(iii) *WNT7A* is the most strongly expressed WNT gene preferentially expressed by tumor cells (Fig. 4c). Other ligands of the network include *WNT2B*, *WNT5A*, and *WNT9A*, differentially expressed by tumor cells and TAMs. These ligands include inducers of both canonical and non-canonical WNT signaling [52]. The canonical pathway depends on both frizzled receptors (*FZD*) and LRP coreceptors, whereas non-canonical signaling does not require LRPs. As multiple LRP genes are expressed by tumor cells and TAMs (Fig. 4c), canonical WNT signaling would be functional in both cell types.
(iv) Multiple *S100* genes are highly expressed in tumor cells and/or TAMs, including *S100A8* and *S100A9* (Fig. 4d). S100A8 and S100A9 proteins interact with surface receptors either as monomers with advanced glycation end products receptor RAGE (*AGER*) and TLR4 or bind as heterodimers to different scavenger receptors [53], all of which are expressed by TAMs (*MSR1*, *SCARA/B, CD36*). Taken together with the particular high expression of both genes in TAMs, these findings point to a pivotal role for TAMs in generating and processing S100A8/A9-associated signals, which also applies to S100A12. Tumor cells express scavenger receptor genes, but not *AGER* and *TLR4* at significant levels, suggesting that these cells are primarily targeted by S100A8/A9 heterodimers. On the other hand, tumor cells but not TAMs express *ERB2*, encoding a receptor for S100A4, suggesting a tumor-selective effect. In contrast, multiple S100 members of varying cellular origins seem to target preferentially TAMs, as suggested by the lack of RAGE expression by tumor cells.
(v) Both tumor cells and TAMs express multiple semaphorins and their receptors (plexins and neuropilins), thereby establishing autocrine as well as paracrine signaling mechanisms (Fig. 4e). While *SEMA3F*, *5A*, *6A*, and *6D* expression is clearly higher in tumor cells, the opposite is true for *SEMA4A* and *6B*. The semaphorin receptor genes *PLXNA1*, *PLXNA3*, *NRP1*, and *NRP2* are expressed by both cell types, whereas *PLXNB1* and *PLXND1* expression is selective for tumor cells and TAMs, respectively.
(vi) Ephrins are also part of the signaling network, with tumor cells playing a major role (Fig. 4f). Thus, tumor cells are the main origin of six different ephrin family members, compared to one subtype expressed by TAMs. Likewise, A-type receptor expression is restricted to tumor cells and B-type receptor expression is considerably higher in, or selective for, tumor cells, the latter exemplified by *EPHB3* and *EPHB4*.
(vii) TAMs play a major role both as producers and targets of multiple chemokines of the *CCL* family (Figs. 2a and 4g). Thus, TAMs preferentially express multiple *CCL* genes, with *CCL2*, *CCL3*, and *CCL4* being the most strongly expressed ones. Moreover, significant expression of receptor genes for these cytokines (*CCR1*, *CCR2*, *CCR5*) was detected only in TAMs. In contrast, several *CXCL* type chemokine genes are expressed by both cell types, however, significant expression of genes coding for their cognate receptor genes was only detectable for *CXCR4* in both cell types, consistent with its description as an independent predictor of a poor clinical outcome of ovarian cancer [54].
(viii) Our study also predict a number of other pathways known to play important roles in tumor progression (Figs. 2 and 4h, i). These include: (1) stimulation of the MET receptor on tumor cells by TAM-produced HGF; (2) the interaction of amphiregulin (*AREG*) produced by both cell types with ERB2, 3, and 4 receptors on tumor cells; (3) the activation of NOTCH receptors on both cell types by JAG1/2 ligands, mainly produced by tumor cells; (4) PDGF signaling by all different family members via PDGFR-A on both cell types; (5) IGF1/2 signaling particularly through IGFR2; and (6) the interaction of angiopoietin-like 4 (*ANGPTL4*) with integrin β1 (*ITGB1*).

### Expression of signaling components in tumor cells from subsets of patients

A number of genes encoding protein mediators were uniformly expressed by tumor cells and/or TAMs (e.g. *IL8*, *KITLG*, *LEP*), but median expression of the corresponding receptor genes was extremely low in both cell types (Figs. 2 and 4; Additional file 3: Datasets S2–S5). Likewise, several receptor genes (e.g. *IL4R*, *INFAR/INFGR*, *PTCH/SMO*) were consistently expressed by tumor cells and/or TAMs, but ligand expression was not detectable. This may be due to the expression of the “missing” ligands and receptors by other host-derived cells or by tumor cell subsets not present in ascites. On the other hand, some of these genes may not be part of the common network due to a restricted expression in smaller subsets of patients. Such genes may be of particular interest, since their expression could be related to the aggressiveness of the disease and thus to its clinical outcome.

We therefore searched for genes not found in the common network but potentially complementing this in a small subfraction of patients. These genes had to fulfill two conditions: (1) TPM >3 in n ≥2 tumor cell or TAM samples (but below the 65 % quantile used in Fig. 2); and (2) coding for proteins representing ligands or receptors for the pathways constructed in Fig. 4. Genes identified by this approach in tumor cells (n = 35; Table 1) and TAMs (n = 14; Additional file 4: Table S4) may indeed be of high relevance, as they code for components of chemokine, TGFβ/BMP, FGF, ephrin, semaphoring, and WNT pathways. We also found the gene coding for norrin (*NDP*), a frizzled 4 ligand unrelated to the WNT family [55], to be expressed in tumor cells from a subset of patients (Table 1).

**Table 1:** Patient-specific expression of cytokine and receptor genes by tumor cells complementing the signaling networks constructed in Figs. 4 and 6

Gene	Description	Min. TPM	Max. TPM
Cytokines			
*BMP8B*	Bone morphogenetic protein 8b	0.32	32.17
*CXCL6*	Chemokine (C-X-C motif) ligand 6	0.00	7.74
*CXCL14*	Chemokine (C-X-C motif) ligand 14	0.00	5.74
*DKK1*	Dickkopf WNT signaling pathway inhibitor 1	0.00	7.49
*EFNA3*	Ephrin-A3	0.95	16.74
*EGF*	Epidermal growth factor	0.09	10.16
*FGF2*	Fibroblast growth factor 2 (basic)	0.02	29.35
*FGF9*	Fibroblast growth factor 9	0.08	15.31
*FGF11*	Fibroblast growth factor 11	0.48	6.17
*FGF13*	Fibroblast growth factor 13	0.08	9.04
*FGFBP1*	Fibroblast growth factor binding protein 1	0.00	28.97
*KITLG*	KIT ligand	0.04	7.48
*NDP*	Norrin (Norrie disease pseudoglioma)	0.00	5.44
*NRG1*	Neuregulin 1	0.02	5.23
*NRG2*	Neuregulin 2	0.04	4.59
*NRG3*	Neuregulin 3	0.00	13.39
*PDGFD*	Platelet derived growth factor D	0.29	6.58
*RSPO3*	R-spondin 3	0.02	8.90
*S100A7*	S100 calcium binding protein A7	0.00	4.03
*S100P*	S100 calcium binding protein P	0.00	12.00
*SEMA3D*	Semaphorin 3D	0.00	6.07
*SEMA3E*	Semaphorin 3E	0.13	93.32
*SEMA4G*	Semaphorin 4G	0.16	6.74
*SEMA5B*	Semaphorin 5B	0.03	19.94
*SEMA6C*	Semaphorin 6C	0.68	4.76
*SEMA7A*	Semaphorin 7A	0.26	9.55
*SFRP1*	Secreted frizzled-related protein 1	0.00	527.58
*TGFB3*	Transforming growth factor, beta 3	0.07	4.51
Cytokine receptors			
*AGER*	Advanced glycosylation end product receptor	0.30	5.01
*EPHA6*	EPH receptor A6	0.13	25.52
*FGFR4*	Fibroblast growth factor receptor 4	0.33	3.76
*FZD2*	Frizzled family receptor 2	0.22	6.18
*FZD10*	Frizzled family receptor 10	0.00	7.95
*IL10RA*	Interleukin 10 receptor, alpha	0.00	5.91
Lipid mediators			
*ALOX15B*	Arachidonate 15-lipoxygenase, type B	0.06	8.03
Lipid receptors			
*LTB4R2*	Leukotriene B4 receptor 2	1.11	3.78
*PTGER3*	Prostaglandin E receptor 3 (subtype EP3)	0.10	11.87

### Identification of a common transcriptome-based signaling network of lipid mediators between tumor cells and TAMs

Lipids derived from phospholipids represent another major group of soluble mediators in ovarian cancer ascites. These comprise mainly breakdown products of phospholipids and metabolites of polyunsaturated fatty acids (PUFAs), in particular AA-derived [30] products of the cyclooxygenase and lipooxygenase pathways [33]. While the first group of mediators, including lysophosphatidic acid (LPA) and PUFAs, is mostly generated by secreted phospholipases, eicosanoid metabolites of the second group are produced exclusively intracellularly. We therefore focused our attention on proteins generating signaling compounds of either group and their receptors and performed an analogous study as described above using datasets of 93 genes encoding enzymes, accessory proteins (Additional file 3: Dataset S6; n = 69), or lipid receptors (Additional file 3: Dataset S8; n = 24).

**Fig. 5:**
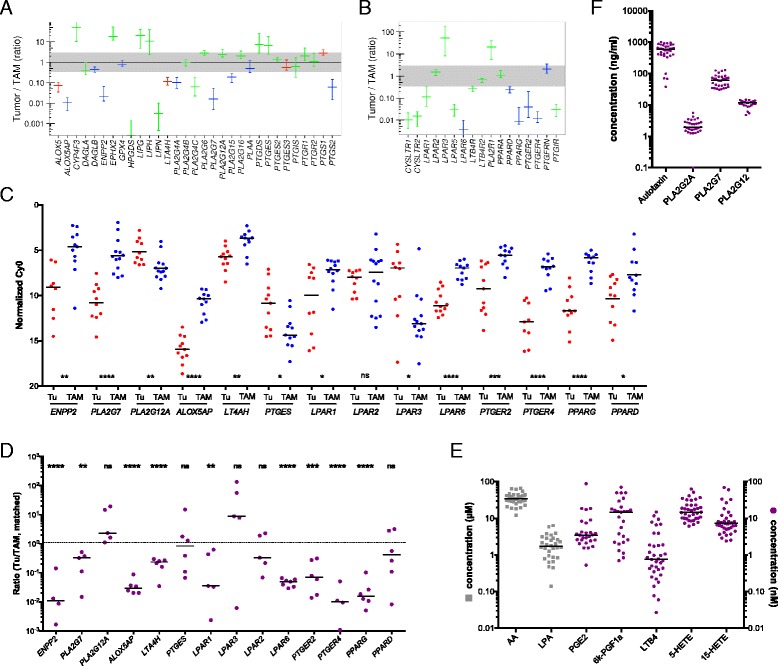
Genes coding for components of lipid signaling expressed in ovarian cancer cells and/or TAMs (RNA-Seq). **a**, **b** Genes coding for enzymes involved in the generation lipid mediators and their receptors. Values represent the ratio of expression in tumor cells versus TAMs (median and 95 % CI). Color code as in Fig. 2. Detailed results are summarized in Additional file 3: Datasets S6-S9. **c** Validation of RNA-Seq data by RT-qPCR of tumor cell samples (each *dot* represents a different sample). **d** RT-qPCR analysis of matched tumor cell and TAM samples from the same patients (each *dot* represents a matched pair). Data are represented as the ratio of expression in tumor cells and TAMs. The *dotted line* indicates a ratio of 1. Data in (**c**) and (**d**) were statistically analyzed by Student’s *t*-test (two-sided, unequal variance). **e** Concentrations of lipid mediators in the ascites fluid from ovarian cancer patients determined by LC-MS/MS. Each *dot* represents a different patient, *horizontal lines* indicate the median. **f** Concentrations of autotaxin (*ENPP2*) and PLA_2_ isoforms in ascites quantified by ELISA

The RNA-Seq data summarized in Fig. 5a and Additional file 3: Datasets S7 and S9 identified 31 genes involved in the enzymatic generation of lipid mediators and expressed in ovarian cancer cells and/or TAMs. Figure 5b shows the data for expression of the corresponding receptor genes (n = 17). A number of key observations were confirmed by RT-qPCR analysis of a larger number of clinical samples (Fig. 5c, d).

We also investigated whether genes expressed at higher levels in tumor cells or TAMs only from a small subfraction (n ≥2) of patients participate might also participate in lipid-mediated signaling pathways. This analysis identified three genes expressed in tumor cells, i.e. *ALOX15B*, the leukotriene B_4_ receptor gene *LTB4R2* and the PGE_2_ receptor gene *PTGER3* (Table 1).

These findings point to a network of lipid mediators established by both tumor cells and TAMs, involving several distinct groups of signaling molecules, as described below.

**Fig. 6:**
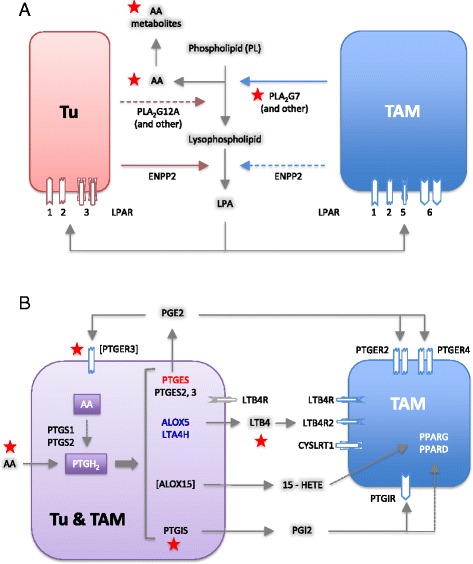
Common lipid signaling in the ovarian cancer microenvironment. **a** A *transcriptome-derived model* depicting the cellular origins and targets (tumor cells, TAMs) of phospholipid degrading enzymes, AA and LPA. **b** An *analogous model* for AA-derived eicosanoid mediators and the sources of enzymes involved in their synthesis. The models are based on the data in Fig. 5a and b. Genes in *square brackets* are expressed in tumor cells in small subset of patients (Table 1). The source of ligands is indicated as follows: *red* for tumor cells, *blue* for TAMs, and *purple* for both. Colored gene names indicate higher expression tumor cells (*red*) or TAMs (*blue*). Each receptor is represented by one or more identical symbols according to their expression levels (as in Fig. 4). [ ]: expressed in subset of patients. *Red asterisks* denote components associated with a poor clinical outcome (based on the data in Figs. 7–9). Gene names are explained in Additional file 3: Datasets S8 and S9
(ii) The second network is established by prostanoids (Fig. 6b), in particular prostanglandin E_2_ (PGE_2_) and PGI_2_ (prostacyclin), both found at substantial levels in ascites (Fig. 5e; 6k-PGF1a is the stable degradation product of PGI_2_), as previously described [56]. Most genes encoding the enzymes required for their synthesis (cyclooxygenases and prostaglandin synthases) are expressed at similar levels by both cells types (*PTGS1*, *PTGES2/3*, *PTGIS*; Fig. 5a, c, d), whereas *PTGS2* is selective for TAMs. A major target of their products seem to be TAMs, which express considerable higher levels of the PGE_2_ and PGI_2_ receptor genes *PTGER2*, *PTGER4*, and *PTGIR* (Fig. 5b, c) with the exception of *PTGER3* expressed only by a small subset of tumor cells (Table 1). In addition, TAMs also show a higher expression of *PPARD* (Fig. 5b–d), encoding the nuclear receptor PPARβ/δ, a possible target for PGI_2_ [57]. Figure 6b shows a schematic representation of these results.
(iii) Products of the lipoxygenase pathway, i.e. 5-HETE, 15-HETE and leukotriene A_4_ (LTA_4_) represent the third network (Fig. 6b). These AA metabolites are present in ascites at readily detectable concentrations (Fig. 5e; LTB_4_ is a stable metabolite of the unstable LTA_4_). This is consistent with the expression of the corresponding lipoxygenase (*ALOX5*), 5-lipoxygenase activating protein (*ALOX5AP*), and leukotriene synthase (*LTA4H*) genes (Fig. 5a, c) in TAMs. In contrast, TAMs also preferentially express the LTB_4_ surface receptor genes *LTB4R*, *LTB4R2*, and *CYSLRT1*/2. 15-HETE has been described as a ligand for the nuclear receptors PPARγ [58] and PPARβ/δ [59], which are both expressed at higher levels in TAMs (Fig. 5b–d). The gene coding for the presumptive 5-HETE receptor *OXER1* [60] is expressed at very low levels in both cell types, if at all (Additional file 3: Dataset S8), suggesting that 5-HETE is more likely to act as a precursor of LTA_4_ in these cells.

(i) The first network is based on products of phospholipid hydrolysis that are generated by specific phospholipases (Figs. 5 and 6a). This conclusion is consistent with the presence of high levels of LPA, AA, specific A2-type phospholipases (in particular PLA_2_G7), and autotaxin in ascites (Fig. 5f). TAMs seem to play an essential role in this context, since they express *PLA2G7* and *ENPP2* at higher levels than tumor cells (Fig. 5a, c). Importantly, the protein levels of 3 phospolipases (PLA_2_G2, PLA_2_G7, and PLA_2_G12A) measured in ascites fluid (Fig. 5f) are consistent with mRNA expression levels in tumor cells and TAMs (Fig. 5a; Additional file 3: Dataset S6). LPA in ascites apparently targets tumor cells and TAMs via specific receptors, since *LPAR1* and *LPAR2* are expressed at similar levels by both cell types, *LPAR3* is selective for tumor cells, *LPAR5* and *LPAR6* for TAMs (Fig. 5b–d). AA is taken up by tumor and host cells [56], where it can regulate signaling pathways, either directly or after metabolic conversion.

### Association of mediator concentrations with clinical outcome

**Fig. 7:**
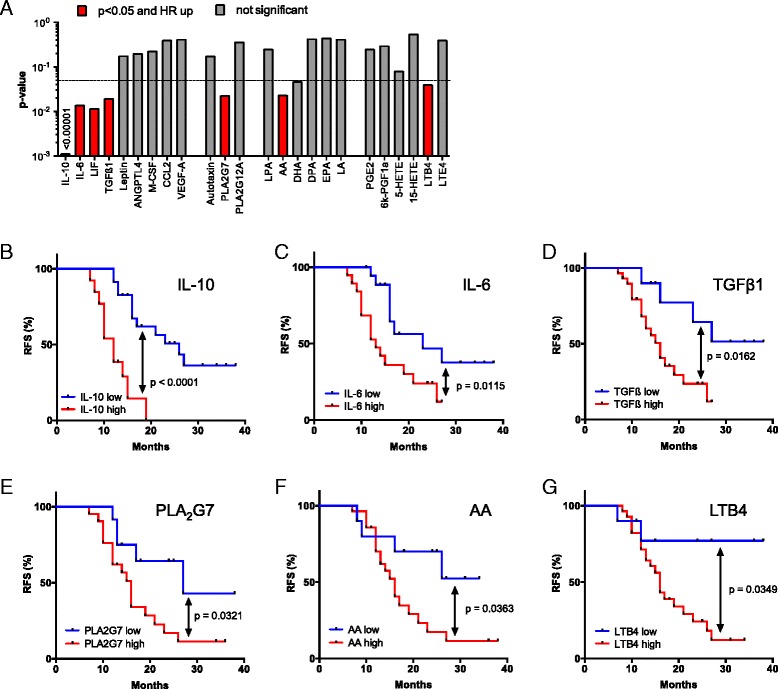
Association of RFS with the levels of cytokines and lipid mediators in ovarian carcinoma ascites. **a** Summary of RFS analyses showing the *p* values determined by Mantel-Cox log rank test. Patients were dichotomized into high and low expressing groups according to the following quantiles (best-fit) and number or patients: IL-10, Q = 0.66 (n = 36); IL-6, Q = 0.5 (n = 39); TGFβ1, Q = 0.25 (n = 39); AA, Q = 0.25 (n = 38); LTB_4_, Q = 0.25 (n = 38); PLA_2_G7, Q = 0.33 (n = 33). Significant instances with a HR >1 are shown in *red*; *grey bars* indicate lack of significant associations. Significance was defined as logrank *p* <0.05 and *p* < Benjamini-Hochberg critical value for false discovery rate (FDR) = 0.10. **b**–**g** Kaplan–Meier plots showing the RFS of patients with high or low ascites levels (best-fit) of IL-10, IL-6, TGFβ1, PLA_2_G7, AA, and LTB_4_ (see “Methods” for details)

We next asked whether mediators in the tumor microenvironment are associated with the clinical outcome of high-grade serous ovarian carcinoma. We therefore assessed potential associations of the ascites levels of cytokines and lipids prior to first-line therapy with RFS by Kaplan–Meier analysis (see Additional file 4: Table S3 for patient-specific clinical features). The logrank *p* values depicted in Fig. 7a demonstrate a clear association of the STAT3-inducing cytokines IL-10, IL-6, and LIF with early relapse (Fig. 7a–c), with IL-10 being the strongest indicator of a poor outcome (*p* <0.0001; logrank hazard ratio [HR] = 4.54; 95 % confidence interval [CI] = 4.56–40.5; median survival 12.0 versus 26.0 months), which is in agreement with a previous study of a smaller cohort of patients [7]. The present study identified inverse associations with RFS for four additional mediators, i.e. TGFβ1, PLA_2_G7, AA, and its metabolite LTB_4_ (Fig. 7a, d–g). In contrast, PLA_2_G12A, autotaxin, and the PLA_2_/autotaxin product LPA did not show any correlation (Fig. 7a). Likewise, the AA metabolites PGE_2_, PGI_2_, 5-HETE, and 15-HETE, also components of the lipid signaling network identified above, were not linked to RFS.

**Fig. 8:**
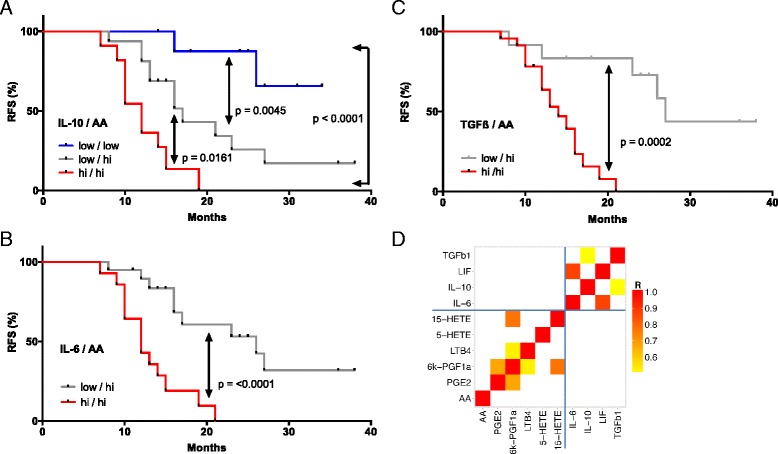
Synergistic association of RFS with the levels of AA and cytokines in ovarian carcinoma ascites. **a**–**c** Patients were trichotomized for RFS analysis, using the best fit thresholds determined in Fig. 7: group 1, cytokine and AA high; group 2, one high/one low; group 3, both low. See “Methods” for details. **d** Pearson correlation matrix for soluble mediators in ovarian cancer ascites shown to be of particular relevance in the present study. The *heatmap* depicts different levels of positive correlation (*red*: high, *yellow*: low, corresponding to a range of R = 0.5–1.0)

The relevance of these cytokines and AA as indicators of an adverse clinical outcome became particularly evident when we determined the RFS for combinations of these mediators. Thus, patients with a high level of either IL-10 and AA, IL-6 and AA, or TGFβ and AA showed a clearly worse outcome compared to those with a high concentration for only one mediator (red versus gray curves in Fig. 8a–c; *p* = 0.016 for IL-10; *p* <0.0001 for IL-6; *p* = 0.0002 for TGFβ). For IL-10, a similar difference was observed between patients showing a high concentration for either IL-10 or AA versus those with low levels of both mediators (Fig. 8a; *p* = 0.0045). A similar analysis for the other two cytokines was not possible due to an insufficient number of cases in the “both low” group. A striking association was observed when patients were compared with high IL-10 and high AA levels to those with low concentrations of both mediators (Fig. 8a; *p* <0.0001; logrank HR = 9.50; 95 % CI = 4.38–47.3; median survival 12.0 versus >34 months).

Pearson analysis revealed low correlation coefficients (r) when cytokine levels were compared to lipid concentrations (Fig. 8d), indicating that the observed clinical associations are not simply a consequence of their co-synthesis. Likewise, the concentrations of AA did not correlate with any of the AA metabolites tested. In contrast, IL-6 and LIF levels were highly correlated (R = 0.87), pointing to common regulatory pathways.

### Association of gene expression levels with clinical outcome

Finally, we sought to establish clinical correlations with components of the common signaling network established above (Fig. 4). Toward this end, we made use of published microarray results for 1018 high-grade serous ovarian cancer patients with documented RFS [38]. The samples used for these analyses were derived from solid tumor masses and therefore contained variable amounts of host-derived cells, including TAMs, as confirmed by the large range of expression values observed for macrophage marker genes across this cohort. Kaplan–Meier analysis for these genes actually showed a clear association of RFS with the expression of these genes (Additional file 2: Figure S4), presumably reflecting the known adverse effect of TAM infiltration on the clinical outcome. In addition, this scenario means that genes not primarily expressed in tumor cells cannot be faithfully analyzed, since it is not possible to separate effects of gene expression from host cell “contamination” in the sample and the algorithm developed in the present study for RNA-Seq cannot be applied to microarrays.

**Fig. 9:**
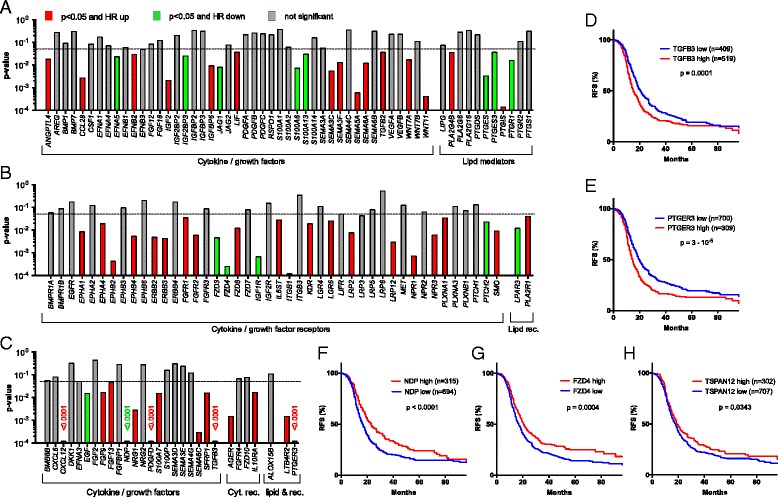
Association of RFS with the expression of genes coding cytokines, growth factors, and lipid mediators or their receptors. Panels (**a**)–(**c**) represent summaries of RFS analyses for 1018 serous ovarian carcinoma patients depicting the results of logrank P tests and the directions of the respective hazard ratio (HR), using the KM plotter database [38]. **a**, **b** Survival analysis for genes in Fig. 2 with an expression ratio (tumor cell/TAM) >0.3. Genes missing from the microarray datasets used by KM Plotter were not included in panels (**a**)–(**c**). Significant instances (for “JetSet best probe”) are shown in *red* (HR >1) or *green* (HR <1); *gray bars* indicate lack of significant associations (*p* ≥0.5) or *p* > Benjamini-Hochberg critical value for FDR = 0.10. Significance was determines as in Fig. 8. **c** Survival analysis as before, but for genes expressed only in small subgroups of patients (see Table 1 and Additional file 3: Dataset S1 ). **d**–**h** Kaplan–Meier plots analyzing the RFS of patients with high or low levels of *TGFB3*, *PTGER3*, *NDP*, or *TSPAN12A* expression. See “Methods” for details

We therefore decided to focus our survival analysis on genes expressed at a higher level in tumor cells relative to TAMs (i.e. more than twofold in Fig. 2). We identified multiple mediator and receptor genes that are clearly (*p* <0.01) associated with a shorter RFS (red in Fig. 9a, b), consistent with their established or suspected functions in tumor progression. These include the cytokine genes *CCL28*, *IGF2*, *SEMA5A*, and *WNT11*, and the receptor genes *EPHB2*, *ERBB2* and *3*, *FGFR2*, *ITGB1*, *LRP12* as well as *NPR1* and *3* (Fig. 9a, b). We also found a surprising association of a favorable clinical outcome with WNT receptor frizzled 4 (*FZD4*) gene expression (Fig. 9a). We performed an analogous survival analysis for genes associated with lipid signaling and expressed at higher levels by tumor cells relative to TAMs (rightmost genes in Fig. 9a, b), based on the data in Fig. 5a and b. A particularly strong association with an adverse clinical outcome was observed for *PTGIS* (*p* = 0.0005), which codes for prostaglandin I_2_ (prostacyclin) synthase (Fig. 6b).

Finally, we performed Kaplan–Meier analyses (Fig. 9d–g) of genes expressed only in small subgroups of our patients (Table 1). A very strong adverse effect on RFS (*p* = 0.0001) was seen with *TGFB3* (Fig. 9c, d), in line with the central role of the associated signaling pathways in cancer, and with *PTGER3* (Fig. 9c, e; *p* <0.0001), encoding a prostaglandin E_2_ receptor (Fig. 6b). Strong associations with poor RFS (*p* <0.001) were also seen with *PDGFD* and *SEMA6C*. However, the most intriguing finding was the identification of *NDP* as a powerful indicator of a favorable clinical course (*p* <0.0001; Fig. 9c, f). *NDP* codes for norrin, which interacts with the receptor frizzled 4 [55, 61] and TSPAN12, a signal-amplifying component of the norrin–frizzled 4 complex [55]. This presumably explains the strong association of *FZD4* with RFS (*p* = 0.0004; Fig. 9g) described above. Furthermore, *TSPAN12* was also inversely associated with RFS (*p* = 0.0343; Fig. 9h). Taken together, these findings provide strong evidence for novel tumor suppressor function of norrin–frizzled 4–TSPAN12 signaling in ovarian cancer.

## Discussion

We have defined a tumor cell and macrophage-driven signaling network operating within the environment of ovarian cancer-associated carcinomatosis involving interleukins, chemokines, members of the TGFβ, WNT, S100, semaphorin and ephrin families, the phospholipid breakdown products LPA, and AA as well as AA metabolites. This network is composed of mostly common, but also patient-specific mediators and receptors and includes pathways previously not identified in the context of ovarian cancer or intercellular signaling in the tumor microenvironment (Figs. 4 and 6). We will discuss these pathways in the following sections, in particular with respect to their association with disease progression after first-line therapy.

### STAT3-inducing cytokines

In agreement with the established function of deregulated STAT3 in ovarian cancer [62], IL-10, IL-6, and LIF were confirmed as components of the signaling network established by tumor cells and TAMs (Figs. 3–5). Their cellular origins and target cells clearly support a pivotal role for TAMs within this network, since these cells are the main producers of IL-10, a major source of IL-6 and the predominant target of IL-10, which presumably plays an important role in their protumorigenic conversion. Expression of LIF and its receptor are higher in tumor cells, pointing to a function for this cytokine beyond its proposed function in TAM polarization [18]. The pathways triggered by these cytokines are also directly relevant to progression of the disease as shown by the inverse association of their ascites levels (Fig. 7) with RFS, consistent with previous studies [7, 63, 64]. Taken together, these data clearly confirm a critical role for cytokine-mediated STAT3 deregulation in ovarian cancer by exerting pro-tumorigenic effects on both tumor cells and macrophages and its potential as a drug target [65].

### TGFβ family

Multiple TGFβ family members have previously been associated with ovarian cancer [19, 25, 66]. In agreement with this established knowledge, we identified several components of this signaling system as important constituents of the ovarian cancer microenvironment, with both tumor cells and TAMs as essential contributors (Fig. 4b). This conclusion is strongly supported by the observed clinical correlations. Thus, the ascites concentration of TGFβ1, mainly produced by TAMs, was associated with early relapse (Fig. 7). A similar adverse link was observed between RFS and the expression of *TGFB2* and *TGFB3* genes by tumor cells, with the latter representing one of the strongest indicators of a poor clinical outcome (Fig. 9c, d). These observations are fully compatible with the known functions of TGFβ ligands in tumor progression [67] and immune suppression [68], as well as the adverse effect of *TGFBR2* and phosphorylated SMAD2/3 on survival [66]. Previous studies have also associated BMP2 and BMP4 with ovarian cancer, both of which are expressed at extremely low levels in tumor cells and TAMs (Additional file 3: Dataset S2), which may be explained by the previous identification of ovarian cancer-associated mesenchymal stem cells as a major source of these cytokines [69].

### Frizzled-mediated signaling

WNT signaling is another major signaling mechanism identified in the present study (Fig. 4c). Seven genes encoding inducers of canonical and/or non-canonical WNT signaling [52], most of which were found to be preferentially expressed by tumor cells. Non-canonical WNT signaling is induced by WNT interaction with FZD without involvement of LRP coreceptors and triggers a calcineurin-NFAT pathway. The expression of at least seven *FZD* genes strongly suggests that the non-canonical pathway is operational. The canonical pathway depends on both FZD and LRP proteins and stimulates β-catenin signaling. Nine *LRP* genes are expressed by tumor cells and/or TAMs (Fig. 4c), suggesting that the canonical pathway is functional in both cell types and utilizes cell type-specific receptors. Importantly, we found a strong inverse association of *WNT11* expression with RFS (Fig. 9a), and also confirmed the previously described [70] correlation of *WNT7A* expression with a poor clinical outcome (Fig. 9a).

R-spondins (*RSPO*) and their receptor LGR5 are required for optimal canonical WNT signaling [22], but expression was insignificant in all samples (*LGR5*; Additional file 3: Dataset S3) or was found in tumor cells from a subset of patients only (*RSPO* genes; Table 1). Since *LGR5* has been identified as a stem-cell specific gene in ovarian epithelial cells in mice [21], this pathway may be restricted to tumor cells with stem-like properties, although the role of LGR5 in human ovarian epithelial cells is unclear.

We also found tumor cell selective expression of the *NDP*, *FZD4*, and *TSPAN12* genes (Fig. 4c, f, g), encoding norrin, its receptor frizzled 4, and a norrin signal-amplifying component of the receptor complex, respectively [55], which were linked to colon cancer angiogenesis in a recent study [61]. Intriguingly, we identified *NDP*, *FZD4*, and *TSPAN12* to be associated with a delayed tumor progression, thus pointing to a novel tumor suppressor function of this signaling pathway in ovarian cancer. This finding is puzzling, since norrin shares with canonical WNT ligands the ability to induce β-catenin, generally considered a pro-tumorigenic pathway. In view of the currently very limited knowledge on norrin-mediated signaling, the mechanism underlying a putative function in tumor suppression remains elusive and may involve hitherto unidentified signal transduction events.

### S100 family

S100 proteins play essential roles in tumor growth and progression, chemoresistance, and immune modulation [53]. Several S100 members are secreted or otherwise released in the extracellular space and interact with surface receptors, including the advanced glycation end products receptor RAGE (*AGER*), scavenger receptors (*MSR1*, *SCARA/B* gene products, *CD36*), EGF family receptors and toll-like receptor 4 (*TLR4*), and stimulate multiple signaling transduction pathways, including NFκB and MAP kinases [53]. Our data show that several *S100* genes, i.e. *S100A4*, *S100A6*, *S100A10*, *S100A8*, and *S100A9*, are expressed at very high levels in both tumor cells and TAMs (Fig. 4d). Furthermore, multiple receptors interacting with different S100 proteins or S100A8/A9 heterodimers are expressed by both tumor cells and TAMs (*SCARA/B*, *CD36*), preferentially by TAMs (*AGER*, *MSR1, TLR4*) or by tumor cells (*ERBB2*), pointing to extensive functional interactions between both cell types. Surprisingly, none of the *S100* genes showed an association with early relapse (Fig. 9b), which is in line with the lack of literature data supporting a role for S100 proteins in the clinical progression of ovarian cancer.

### Semaphorins and ephrins

Semaphorins and ephrins, originally identified as axon guidance molecules, also have essential physiological functions during organ development, angiogenesis, and immune regulation [71–73]. More recently, their involvement in cancer cell migration, invasion, and metastasis has been uncovered, but is currently only partially understood. Activation of plexins by semaphorins results in the transactivation of oncogenic receptor tyrosine kinases, including MET, ERBB2, and KDR [73, 74]. Individual family members can be associated with either stimulatory or inhibitory effects on tumorigenesis and angiogenesis. For instance, a potential role in suppression of malignant melanoma has been described for *PLXNB1* [75], while cooperation with ERBB2 and a pro-metastatic role was reported for breast cancer cells [76]. We have identified multiple genes encoding components of both semaphorin and ephrin signaling in both tumor cells and TAMs, i.e. 13 semaphorins and at least six cognate receptors, as well as six ephrin members and seven receptors. These findings point to a complex signaling network established by tumor cells and TAMs (Fig. 4e), involving both autocrine and paracrine signaling mechanisms, as well as cell type-selective expression of ligands and receptors. Five of these genes, *SEMA3C*, *3 F*, *5A*, *6A* and in particular *6C*, are associated with early relapse (Fig. 9a and c). Likewise, four ephrin receptor genes (*EPHA1*, *EPHA4*, *EPHB2*, *EPHB4*) showed an adverse clinical association (Fig. 9b). Our findings therefore strongly support a tumor-promoting role for axon guidance ligands and their receptor in ovarian cancer. As these are expressed by tumor cells as well as TAMs, it is likely that both cell types play a role in this context.

### Chemokines

Chemokines are produced by and target tumor and tumor-associated host cells through a large number of ligand-selective surface receptors, thereby establishing a large intercellular signaling network. These include TAMs [77], but their precise integration into the microenvironment of a human cancer has not been established. Our data support an essential role of TAMs within the chemokine network, since they express 11 *CCL* members (Fig. 2a) and three CCR receptors (Fig. 2b), of which two (*CCL2* and *CCL5*) are also expressed by tumor cells. TAMs also play an important role as producers of ten different chemokines of the CXCL family (Fig. 2a), but express only two *CXCR* receptor genes. One of these is CXCR4, thus confirming the prosed role of the CXCL12–CXCR4 axis in the progression of many tumor types [78], including ovarian cancer [54]. Since chemokines mainly address other cell types, in particular T-cells, the lack of expression of other *CXCR* genes in tumor cells and TAMs is conceivable.

### Phospholipid breakdown products

Tumor cell and TAMs express multiple genes for secreted phospholipases, with *PLA2G7*, preferentially expressed by TAMs, as the major subtype (Fig. 5a). Intriguingly, PLA2G7 ascites levels are associated with a short RFS (Fig. 7a, e), indicating a clinical relevance for the phospholipid breakdown products. These include LPA, generated from lysophospholipids by autotaxin, and PUFAs. Our survival analyses did not show any significant correlation of LPA or autotaxin levels in ascites with the clinical outcome (Fig. 7a). However, the former result must be considered with some caution, since LPA represents a mixture of several compounds with different fatty acids in the sn1 position. It has been shown that different LPA species can exert different biological effects, which may be obscured when these are collectively quantified. Furthermore, according to the manufacturer, the antibody used for this analysis (ELISA) recognizes the minor forms (e.g. linolenic 18:3 LPA) with a higher affinity compared to the more common LPA species (e.g. oleic 18:1 LPA). The relevance of LPA as a potential indicator of early ovarian cancer relapse has therefore to be re-evaluated in future studies using methods that are able to discriminate different LPA species.

On the other hand, a clear inverse association with RFS was observed for AA (Figs. 4, 7a, f). The clinical relevance of AA is strongly supported by our finding that the adverse effect of cytokines, like IL-6, IL-10, and TGFβ were enhanced by the simultaneous presence of high AA levels, pointing to a hitherto unknown cooperation in causing therapy failure and disease progression. Importantly, AA concentrations did not show any significant correlation with IL-6, IL-10, or TGFβ (Fig. 8d), excluding the possibility that the observed clinical correlations are due to a common mechanism regulating the synthesis of these mediators.

### Arachidonic acid metabolites

AA is metabolized to a number of highly bioactive eicosanoid derivatives, in particular cyclooxygenase-derived prostanoids and lipoxygenase-derived HETEs and leukotrienes. In ovarian cancer, several components of these pathways are present in ascites, and the required enzymes are expressed by both tumor cells and TAMs (Fig. 6b). These mediators seem to act primarily on TAMs, including PGE2, PGI2, and 15-HETE, as judged by the expression of their cognate receptors. An exception was LTB_4_ with receptors on both cell types. A clinical relevance of these mediators is suggested by the observed inverse associations of RFS with the ascites levels of LTB_4_ (Figs. 4, 7a, g) and the expression of the *PTGIS* and *PTGER3* genes (Figs. 4, 9b, e), encoding PGI_2_ synthase and a PGE_2_ receptor, respectively (Fig. 6b). These findings could, at least in part, explain the adverse effect of AA on survival, i.e. by serving as a precursor of pro-tumorigenic metabolites.

It can, however, not be excluded that non-metabolized AA contributes to this effect. We have recently shown that PPARβ/δ, which is expressed preferentially in TAMs (Fig. 2b), is deregulated by PUFA ligands in ovarian cancer ascites [56]. It is, however, very unlikely that PPARβ/δ mediates the adverse effect of AA on RFS, because the major ascites-associated PUFA with strong agonistic effect on PPARβ/δ is linoleic acid [56], which, in turn, is not linked to survival at all (Fig. 7a). Even though other targets for non-metabolized AA have been identified [79–82], AA-triggered signaling is poorly understood, making it difficult to speculate on the molecular mechanism underlying the clinical effect discovered in the present study.

## Conclusions

In spite of the clearly documented pivotal role of the tumor microenvironment in tumor growth, progression, and immune escape, the reciprocal interactions of tumor and host cells through soluble mediators are only partially understood. In the present study we have established a global RNA-Seq based strategy to address this problem using tumor cells and TAMs from ovarian carcinoma ascites. As a first step, we developed an algorithm to adjust sequencing data for the presence of contaminating cells in the samples analyzed, i.e. macrophages in tumor cell fractions or vice versa. After optimization on training datasets the algorithm was successfully applied to the ovarian cancer samples used in the present study, indicating that the method should be generally applicable to tackle the problem of contaminating cells in RNA-Seq samples.

Taken together, our observations suggest that the strategy used in the present work is a generally applicable approach to address complex interactions in the tumor microenvironment. These include several important questions not addressed by the current study. First, it is possible that we missed clinically relevant genes, because of the necessity to exclude genes expressed at high levels in TAMs from our survival analysis. Thus, survival-associated receptor genes expressed primarily in TAMs would not have been found. Future sufficiently large RNA-Seq studies of pure cell types or single cells in conjunction with survival analyses will have to answer this question. Second, host cells other than TAMs are clearly important constituents of the tumor microenvironment, but their role within a signaling network are even less understood. In ascites these are primarily other immune cells and mesothelial cells, while fibroblasts and endothelial cells are rare or absent. Thus, the integration of T cells into the signaling network operating among the ascites-associated cells will be an important next step. Third, it is unknown how ascites-associated tumor and host cells differ from their counterparts in solid tumor masses. Purification of cells from metastases of the same patients could be used to address this question, and also to analyze the contribution of host-derived cell types restricted to solid tumor tissue.

## Methods

### Patient samples

Ascites was collected from patients with high grade serous ovarian carcinoma undergoing primary surgery at the University Hospital in Marburg. Written informed consent for the use of ascites for research purposes and publication of the results obtained from this research was obtained from all patients prior to surgery according to the protocols approved by the ethics committee of Marburg University (Az 205/10). Patient characteristics are presented in Additional file 4: Tables S1 and S3. Clinical courses were evaluated by RECIST criteria [83] in patients with measurable disease or profiles of serum CA125 levels [84], according to the recommendations by the Gynecologic Cancer InterGroup (GCIG). Only patients with observations periods ≥12 months after first-line surgery were included in the survival analysis. All experimental methods comply with the Helsinki Declaration.

### Isolation of TAMs from ovarian cancer ascites

Mononuclear cells were isolated from ascites by Lymphocyte Separation Medium 1077 (PromoCell) density gradient centrifugation and further purified by magnetic cell sorting (MACS) using CD14 microbeads (Miltenyi Biotech). TAMs were directly analyzed by FACS as described below or lysed in PeqGold (Peqlab) for RNA preparation.

### Tumor cell/spheroid isolation from ascites

Mononuclear cells were isolated from ascites by Lymphocyte Separation Medium 1077 (PromoCell) density gradient centrifugation. Tumor spheroids were separated by filtration using 30 μm and 40 μm cell strainer (Miltenyi Biotech) resulting in either spheroids of medium size (30–40 μm = “m”) or large size (>40 μm = “L”). Small tumor spheroids (<30 μm = “s”) and tumor single cells (sc) were further purified by depletion of peritoneal leucocytes using CD45 microbeads and magnetic cell sorting (MACS) (Miltenyi Biotech). Purified tumor cells were lysed in PeqGold (Peqlab) for RNA preparation, analyzed by flow cytometry, or cultured for testing of chemoresistance. The purity of tumor spheroids/cells was >90 % EpCAM+ cells, except for sample OC84s (>85 %, Additional file 4: Table S2).

### Characterization of tumor cells/spheroids by flow cytometry

Prior to FACS staining, tumor spheroids were dissociated into single cells by trypsination for 10 min at 37 °C, followed by vortexing for 10 s. To analyze cell cycle distribution, tumor single cells were fixed in 70 % ice-cold ethanol, washed with PBS + 2 % FCS, and treated with 100 μL RNAse (1 mg/mL) at 37 °C for 20 min. Cells were stained with 10 μL propidium iodide (1 mg/mL) for 30 min. FACS analysis was performed on a FACS Canto II instrument using Diva Software (BD Biosciences). Proliferation was analyzed by FACS after staining tumor single cells with anti-Ki67 PEVio770, anti-CD45 FITC, and anti-EpCAM PE antibodies (all Miltenyi Biotech).

### Flow cytometry analysis of ascites-associated cells

Gene expression profiles generated from RNA-Seq datasets were verified in TAMs and tumor cells by FACS analysis. Mononuclear cells from patients’ ascites were simultaneously stained with Vioblue-labeled anti-human EpCAM (Miltenyi Biotech) as tumor marker and FITC-labeled anti-CD14 (Miltenyi Biotech), PE-labeled anti-CD163 (eBioscience), or APC-labeled anti-CD206 (Biozol) as TAM marker. In addition, FITC-labeled anti-TGFbeta RIII and PE-labeled anti-LIF-R (all R&D Systems) were used for surface staining. Intracellular staining of permeabilized cells was performed with APC-labeled anti-IL-8 (eBioscience), FITC-labeled anti-S100A8/A9 (Life Technologies) and FITC-labeled anti-S100A14 (antibodies-online) as described previously [7]. Isotype control antibodies were purchased from BD Biosciences, Miltenyi Biotech, and eBioscience. Cells were analyzed by flow cytometry and results were calculated as percentage of positive cells and mean fluorescence intensities (MFI).

### In vitro testing of chemoresistance

Tumor spheroids or single cells from patients were cultured in M199 media (Life Technologies) plus 10 % autologous, cell-free ascites with or without 10 μM carboplatin (Sigma Aldrich) and 10 nM paclitaxel (AdipoGen) at 37 °C, 5 % CO_2_ (approximately 2.5–5 × 10^5^ cells/mL). After 6 days, the 3-[4,5-dimethylthiazol-2-yl]-2,5-diphenyl tetrazoliumbromid (MTT) assay was performed to assess cell viability as described previously [85]. The percentage of chemoresistant tumor cells in the carboplatin/paclitaxel treated culture was calculated relative to cells treated with solvent control (DMSO).

### Analysis of soluble mediators in cell-free ascites

Soluble mediators in ascites of ovarian cancer patients were quantified using commercial ELISA Kits according to the instructions of the manufacturers. Human IL-6, IL-10, LIF, VEGF-A, CCL-2, and TGFβ1 levels in ascites were analyzed by ELISA kits purchased from eBioscience. ANGPTL4 levels were determined using ELISA kit from Aviscera Bioscience, leptin by ELISA Kit from RayBiotech and LPA by ELISA kit from Echelon. The phospholipase A2, Group XIIA (PLA2G12A) ELISA Kit was from antibodies-online, the PLA2G2A ELISA kit from Biozol, and the ENPP-2/Autotaxin, CSF-1, S100A8, and PLA2G7 ELISAs from R&D Systems.

### Quantification of lipids by liquid chromatography - tandem mass spectrometry (LC-MS/MS)

Ascites samples (1 mL) were spiked with 100 μL deuterated internal standard and extracted using solid reverse phase extraction columns (Strata-X 33, Phenomenex). Fatty acids derivatives were eluted into 1.0 mL of methanol, lyophilized, and resuspended in 100 mL of water/acetonitrile/formic acid (70:30:0.02, v/v/v; solvent A) and analyzed by LC-MS/MS on an Agilent 1290 separation system. Samples were separated on a Synergi reverse-phase C18 column (2.1 × 250 mm; Phenomenex) using a gradient as follows: flow rate = 0.3 μL/min, 1 min (acetonitrile/isopropyl alcohol, 50:50, v/v; solvent B), 3 min (25 % solvent B), 11 min (45 % solvent B), 13 min (60 % solvent B), 18 min (75 % solvent B), 18.5 min (90 % solvent B), 20 min (90 % solvent B), 21 min (0 % solvent). The separation system was coupled to an electrospray interface of a QTrap 5500 mass spectrometer (AB Sciex). Compounds were detected in scheduled multiple reaction monitoring mode. For quantification a 12-point calibration curve for each analyte was used. Data analysis was performed using Analyst (v1.6.1) and MultiQuant (v2.1.1) (AB Sciex).

### RT-qPCR and RNA-Seq

cDNA isolation and qPCR analyses were performed as described [86], using *L27* for normalization and evaluated by the Cy0 method [87]. Primer sequences are listed in Additional file 4: Table S5. RNA-Seq was carried out on an Illumina HiSeq 1500 as described [85]. Summarized read counts are shown in Additional file 3: Dataset S1. Genome assembly and gene model data were retrieved from Ensembl revision 74.

### Sequencing data availability

Sequencing data were deposited at EBI ArrayExpress (accession numbers E-MTAB-3167 and E-MTAB-4162).

### Bioinformatic analysis of RNA-Seq data

RNA-Seq data were aligned to Ensembl v74 using STAR (version STAR_2.4.1a) [88]. Gene read counts were established as read count within merged exons of protein coding transcripts (for genes with a protein gene product) or within merged exons of all transcripts (for non-coding genes). TPM (transcripts per million) were calculated based on the total gene read counts and length of merged exons. Genes were considered expressed if they had a minimum TPM of 3. All genomic sequence and gene annotation data were retrieved from Ensembl release 74, genome assembly hg19. Our full analysis scripts and computational pipeline are available upon request.

### Adjustment of RNA-Seq data for contaminating cells

The development and testing of our algorithm, including benchmarking against other published algorithms, are described in detail in Additional files 1 and 5.

Simulations for Fig. 1a were performed 12,000 times on data retrieved from GSE60424 [51]. The dataset consists of highly purified immune cells from patients with various autoimmune diseases. Samples annotated “whole blood” and sample lib264 were excluded, as the latter showed monocyte contamination. Mixtures were calculated by resampling the larger sample to the size of the smaller one and mixing at a chosen percentage. Reference expressions were calculated from all non-mixed samples of the respective tissues. Contamination estimation and correction was performed as described in detail in Additional file 1.

OC66s, TAM72, and TAT31 were used as reference samples for pure tumor cell, TAM, and TAT populations, respectively (see Fig. 1b, c). The automated procedure selected the following marker genes for adjusting tumor cell datasets:

- TAM marker genes: *AIF1*, *C1QB*, *C1QC*, *CCR1*, *CD36*, *CMKLR1*, *CR1*, *FCGR2A*, *FCGR3B*, *FPR3*, *ITGAM*, *MARCO*, *MPEG1*, *MRC1L1*, *STAB1*, *TLR4*, *VCAN*.
- TAT marker genes: *ATP2A3*, *C16orf54*, *CCR4*, *CCR7*, *CD2*, *CD247*, *CD3E*, *CD96*, *GZMK*, *IL2RB*, *IL2RG*, *KCNA3*, *LEF1*, *NKG7*, *PRF1*, *RHOH*, *ZNF831*.

For adjusting TAM datasets the following marker genes were selected:

- Tumor cell marker genes: *ASS1*, *CDH1*, *CLDN4*, *CT45A1*, *CT45A3*, *CT45A4*, *CT45A5*, *DSP*, *EPCAM*, *ESRP1*, *IGFBP3*, *KRT7*, *LRP6*, *MEIS1*, *PRAME*, *SLPI*, *VTCN1*.
- TAT marker genes: *ATP2A3*, *CAMK4*, *CCR4*, *CD8A*, *CD8B*, *CST7*, *KCNA3*, *KLF12*, *LCK*, *LIME1*, *MT1X*, *NKG7*, *PRF1*, *RHOH*, *RLTPR*, *TCF7*, *TGFBR3*.

The source code for implementing our algorithm and the simulations described in the present study are included as Additional file 6 and deposited at GitHib (https://github.com/IMTMarburg/rnaseqmixture) and Zonodo (doi:10.5281/zenodo.48872).

### Statistical analysis of experimental data

Comparative data were statistically analyzed by Student’s *t*-test (two-sided, unequal variance) using GraphPad Prism 6.0. Results were expressed as follows: *p <0.05; **p <0.01; ***p <0.001; ****p <0.0001. CIs were calculated using the bootstrap method.

### Survival-associated gene expression analysis

Associations between gene expression and relapse-free survival of ovarian cancer patients were analyzed using the web based tool “KM Plotter” [38] (http://kmplot.com) with the following settings: “auto select best cutoff,” probe set option: “JetSet best probe,” histology: serous, datasets: all; other settings: default). The 2015 version of KM Plotter used contains the following 13 datasets: GSE14764 (n = 80), GSE15622 (n = 36), GSE18520 (n = 63), GSE19829 (n = 28), GSE23554 (n = 28), GSE26193 (n = 107), GSE26712 (n = 195), GSE27651 (n = 49), GSE30161 (n = 58), GSE3149 (n = 116), GSE51373 (n = 28), GSE9891 (n = 285), TCGA (n = 565). The GraphPad Prism software was used to analyze associations of soluble mediator concentrations in ascites fluid with RFS (Kaplan-Meier plots, logrank *p* values, logrank HR, and median survival times). Multiple hypothesis testing was accounted for out by controlling the FDR using the Benjamini-Hochberg method.

## Additional files

Additional file 1: Description and optimization of the algorithm and benchmarking against published methods. (PDF 5117 kb) Additional file 2: Supplementary Figures S1–S4. (PDF 946 kb) Additional file 3: Datasets S1–S7. (XLS 26437 kb) Additional file 4: Supplementary Table S1–S5. (XLS 48 kb) Additional file 5: Source code. (TXT 9 kb) Additional file 6: Assembly of gene sets. (PDF 200 kb)

## Abbreviations

AA: arachidonic acid
ChIP: chromatin immunoprecipitation
CI: confidence interval
ELISA: enzyme-linked immunosorbent assay
FDR: false discovery rate
HR: hazard ratio
LPA: lysophosphatitic acid
LC-MS/MS: liquid chromatography - tandem mass spectrometry
LT: leukotriene
MAE: mean absolute error
PG: prostaglandin
PUFA: polyunsaturated fatty acid
RNA-Seq: RNA sequencing
RFS: relapse-free survival
TAM: tumor-associated macrophage
TAT: tumor-associated lymphocyte
TPM: transcripts per million
