# Supplementary material for: A transcriptome-based global map of signaling pathways in the ovarian cancer microenvironment associated with clinical outcome
Source: Genome Biol. 2016 May 23;17:108. doi: 10.1186/s13059-016-0956-6 (PMC4877997; doi:10.1186/s13059-016-0956-6)
Supplement: Additional file 1: — Description and optimization of the algorithm and benchmarking against published methods. (PDF 5117 kb) [file 13059_2016_956_MOESM1_ESM.pdf]

Algorithms for deconvoluting different cell types from expression data sets: applicability to the adjustment of RNA-Seq data of ovarian cancer associated cells

Background

Enrichment of specific cell types from ovarian cancer associated ascites is often faced with the problem of other "contaminating" cell types. Determining such contaminations from gene expression profiles *in silico* is a well-established problem commonly referred to as '"deconvolution" (see a recent review of available algorithms [1]). Once the composition of a sample has been established, a correction against the contamination can be implemented.

We found that none of the available algorithms is suitable for our specific conditions:

(i) we are dealing with RNA-Seq data, while many older algorithms have been established on micro array data,

(ii) we have a relatively small number of samples to correct and learn from,

(iii) our datasets reflect two or three cell types involved, which are highly dissimilar,

(iv) there is no prior knowledge of appropriate marker genes (since TAMs are not canonically activated macrophages,

(v) the profile of tumor cells in ascites was undetermined prior to the present study, and

(vi) we require both an estimate of the contamination and a correction of expression gene profiles.

Description of algorithm

Our chosen approach is mathematically straightforward: Starting with two pure reference samples representing the cell type of interest ("target") and the contaminating cell type we select a set of suitable contamination marker genes, use these to estimate the extent of contamination and then adjust the target dataset by a linear model. The purity of reference samples must be determined by other methods, e.g. microscopy or flow cytometry.

Potential marker genes are defined as genes with (i) at least a three fold change between target and contaminating cell types and (ii) a maximum expression of 10 TPM in non-target cell types. These candidates are ranked by fold change, the top j are skipped (see below) and a fixed number is chosen.

Expression of marker genes is modeled as

$$y_{observed} = y_{contamination} * p + y_{target} * (1 - x)$$

with y_S being gene expression in TPM and x the contamination percentage of a single contamination. We replace $y_{target} * (1 - x)$ with the expression in our target cell type reference sample ($y_{reference}$), thereby introducing a slight bias to underestimate the contamination percentage. Note that for marker genes, y_{target} is less than 10 TPM, while $y_{contamination}$ is typically much larger. An underestimation of the contamination keeps our correction conservative, preventing too harsh a correction.

Our final estimation (P) is the median of

$$p = \frac{(y_{observed} - y_{reference})}{y_{contamination}}$$

x smaller than 0 is replaced by 0, P >1.0 is rejected.

To correct, for each gene we replace $y_{observed}$ with

$$\frac{y_{corrected} = (y_{observed} - P * y_{contamination})}{(1.0 - P)}$$

thereby rescaling to TPM.

To extend the approach to a three-cell line setting, we estimate contamination percentages for each cell type independently using disjunct mark sets and replace $y_{observed}$ with

$$y_{corrected} = \frac{y_{observed} - P_1 * y_{contamination1} - P_2 * y_{contamination2}}{1.0 - P_1 - P_2}$$

Implausible results ($P_1 + P_2 > 1.0$) are rejected.

Estimation of nuisance parameters

The algorithm has two nuisance parameters, the number of genes to choose (k), and the number of ranks to skip (j). Nuisance parameters were optimized in a simulation setting with 1,000 repetitions per parameter value. Monocyte samples (contamination) from GSE60424 (Table 1) were mixed with samples from other blood cells (target) at randomized percentages. One monocyte and one target sample (not part of the mixture) were chosen as reference. It was found that no straightforward correlation between the nuisance parameters and the accuracy of the algorithm exists (Figure 1).

Table 1: Samples in dataset GSE60424

Tissue	Sample count	**Comment**
B-Cells	20	
CD4	20	
CD8	19	Sample lib264 omitted due to monocyte signal
NK	14	
Monocytes	20	
Neutrophils	20	
Whole blood	20	

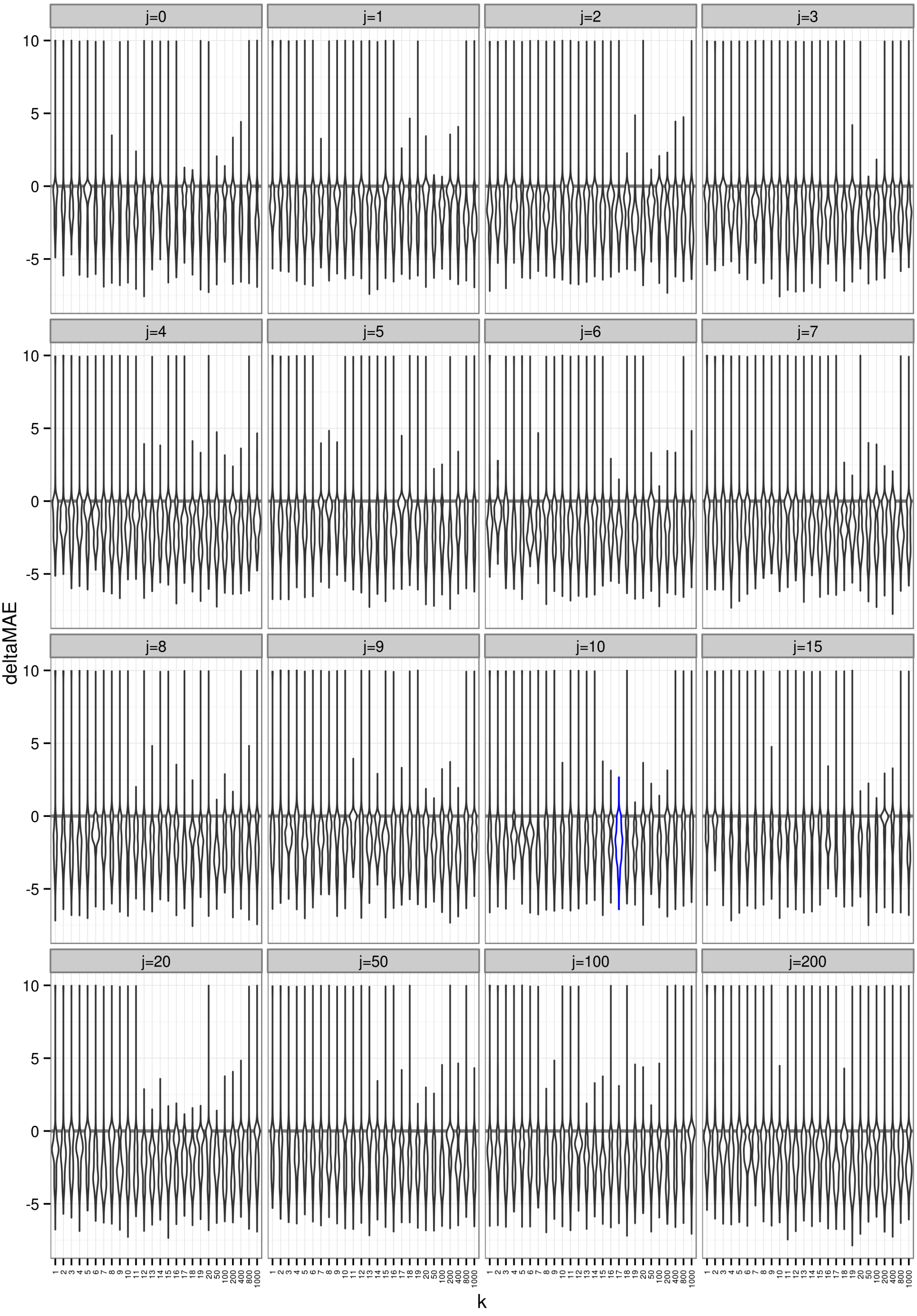

Figure 1: Parameter sweep. Shown is the change in mean absolute error between (corrected) mixture TPM and ground truth. We performed 500 simulations per data point. Blue: values chosen for correction in the main paper.

Evaluation of algorithms

We evaluated algorithms in a simulation setting, in which arbitrary percentages of randomly chosen samples of different tissues were mixed. Different samples from the same tissue were chosen as references. Two RNA-Seq data sets of different tissues were used: the large Gene Tissue-Expression (GTEx) dataset [2] (Table 2), and E-MTAB-2836 [3], a smaller dataset that includes immune related tissue (Table 3). Simulations were run 10,000 times.

Table 2: Tissues in GTExdataset
(retrieved on 2015-06-08, only the samples in the GTEx pilot study were used).

Tissue	Sample count
Adipose - Subcutaneous	128
Artery - Tibial	137
Heart - Left Ventricle	95
Lung	133
Muscle - Skeletal	157
Nerve - Tibial	114
Skin - Sun Exposed (Lower	126
Thyroid	120
Whole Blood	191

Table 3: Tissues in E-MTAB-2836 dataset

Tissue	Sample count
adipose tissue	7
bone marrow	8
colon	8
endometrium	9
gall bladder	7
heart	9
lung	8
lymph node	13
placenta	7
prostate	7
small intestine	8
testis	8
thyroid	9

The in-silico mixture allowed evaluation of algorithms on the difference between corrected and uncorrected Mean-Absolute-Error (MAE)

$$\text{deltaMAE} = \text{mean}(|y_{corrected} - y_{groundtruth}|) - mean(|y_{mixture} - y_{groundtruth})|).$$

Comparison with CIBERSORT and DeconRNASeq

We next compared our algorithm with two recently published methods, CIBERSORT and DeconRNASeq.

CIBERSORT [4] was established to distinguish 22 closely related immune cell types via support vector regression from microarray data, although the authors expect it to work with RNA-Seq data. Besides an estimation of the distribution of cell types, it provides a p-value "to test the null hypothesis that no cell types in the signature matrix […] are present in a given GEP [gene expression profile] mixture". The 22 immune cell type signature (LM22) provided with CIBERSORT is unable to estimate macrophage contents in our tumor cell samples according to its own p-value estimation (our most contaminated sample: $p = 0.02$; all other samples: $p > 0.1$).

To generate a custom signature matrix using CIBERSORT's automated procedure three pure samples of each cell type are required. In addition, the CIBERSORT FAQ states "Building the specific collection of genes in a signature matrix is a nuanced process, and is critical for its performance on complex tissues. Construction and validation of LM22 required more than a year of investigation". Consequently, we ran CIBERSORT with ad-hoc signature matrices composed of the 500 genes showing the highest extent of differential expression (250 up, 250 down, min. 10 TPM in the higher tissue).

DeconRNASeq [5] models RNA-Seq samples as linear mixtures estimated via quadratic programing using a signature matrix. The signature matrix captures the expression difference of hundreds of genes across pure samples. The implementation does not offer correction, nor an automated way to build the signature matrix. We build an ad-hoc signature matrix as above for CIBERSORT.

Our algorithm was run $k = 250$, $j = 0$ in order to keep comparable parameters. While our algorithm was able to predict the contamination in most cases ($r = 0.8$), both CIBERSORT and DeconRNAseq ($r < 0.3$) failed using these ad-hoc signature matrices (Figures 2 and 3).

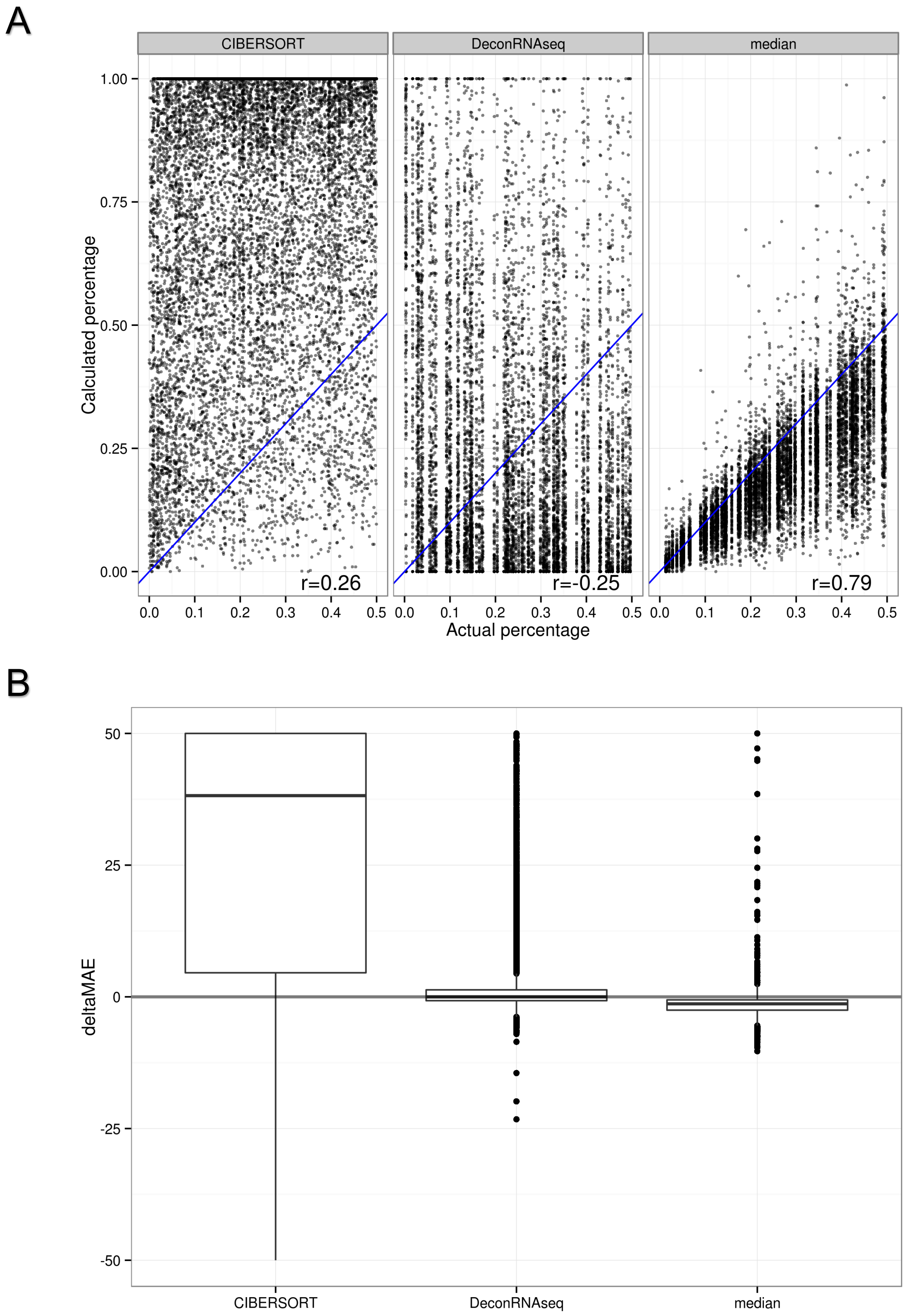

Figure 2: Algorithm comparison with CIBERSORT and DeconRNASeq on GTEx samples [2]. Conditions: 10,000 simulations per algorithm, random percentage between 0 and 50%, single randomly chosen reference per tissue and simulation. **(A)** Actual versus calculated percentage. Blue: diagonal. **(B)** Resulting deltaMAE between corrected and uncorrected mixtures in comparison to the ground truth.

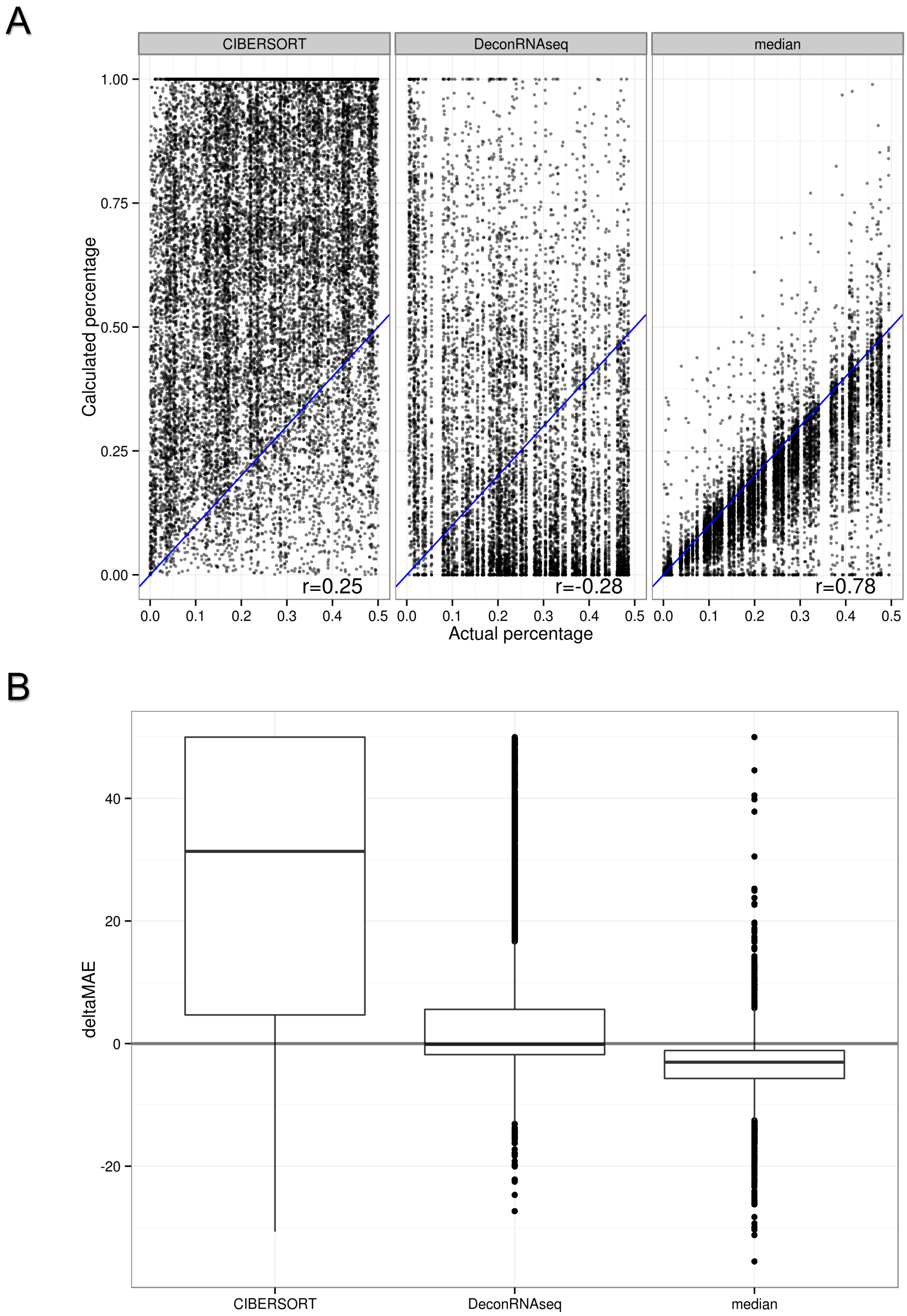

Figure 3: Algorithm comparison on E-MTAB-2836 dataset [3]. Conditions: 10,000 simulations per algorithm, random percentage between 0 and 50%, single randomly chosen reference per tissue and simulation. **(A)** Actual vs calculated percentage. Blue: diagonal. **(B)** Resulting deltaMAE between corrected and uncorrected mixtures in comparison to the ground truth.

Other algorithms

A number of other algorithms were considered, but their application was rejected for technical reasons.

ContamDE's [6] focus is on differential expression between tumor (mixture) and normal (pure) samples. It requires at least two of each and has a long runtime (on the order of minutes), complicating simulations.

UNDO [7] is a completely unsupervised algorithm that merely uses mixture samples to deconvolute tumor and normal tissue. It does not use pure references and determines suitable marker genes solely from the mixture data. Although it was established on microarray data, it has been used with some success on RNA-Seq data [6]. When adjusting our simulation to provide two mixture samples, we found that UNDO only works if both mixtures are mixtures of the same (sample, contamination) samples. This makes it unusable in our setting, where there is only one mixture per patient available.

TEMT [8] works on transcription level RNA-Seq alignments. Transcription level analysis is inappropriate for the cell-cell network investigated in this study.

ESTIMATE [9] produces an 'ImmunoScore' that is not usable for correction.

IsoPure [10] explicitly biases its results to the assumption that the two cell types being deconvoluted are closely related (tumor and normal tissue).

DeMix [11], **Dsection** [12] and **PSEA** [13] have only been established on microarrays.

Limitations of our algorithm

Finally, two important limitations of our approach need to be briefly addressed, although these are not relevant to the present study:

First, our algorithm is unable to distinguish closely related cell types, such as the CD4 and CD8 sample from GSE60424 (Figure 4).

Second, as shown in Figure 5, small numbers of reference sample combinations caused all instances in Figure 3 where the algorithm actually increased MAE. Therefore, the references must be well chosen to represent the contaminating cell types.

Availability

A python implementation of our algorithm is included as Additional File 6.

The code is also available, together with our simulation code, from https://github.com/IMTMarburg/rnaseqmixture

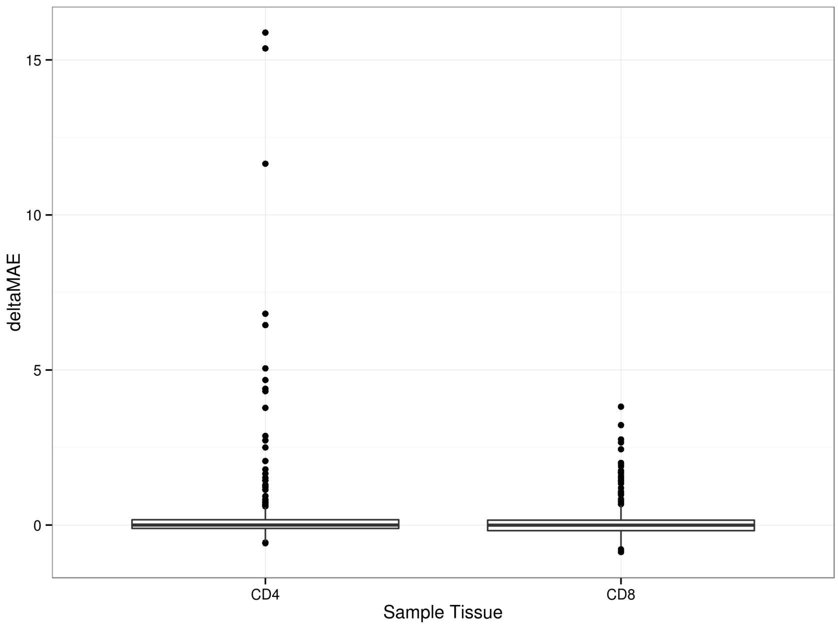

Figure 4: Failure of correction on closely related cell types from GSE604242. 10,0 simulations. CD4 samples were contaminated with CD8 and *vice versa.*

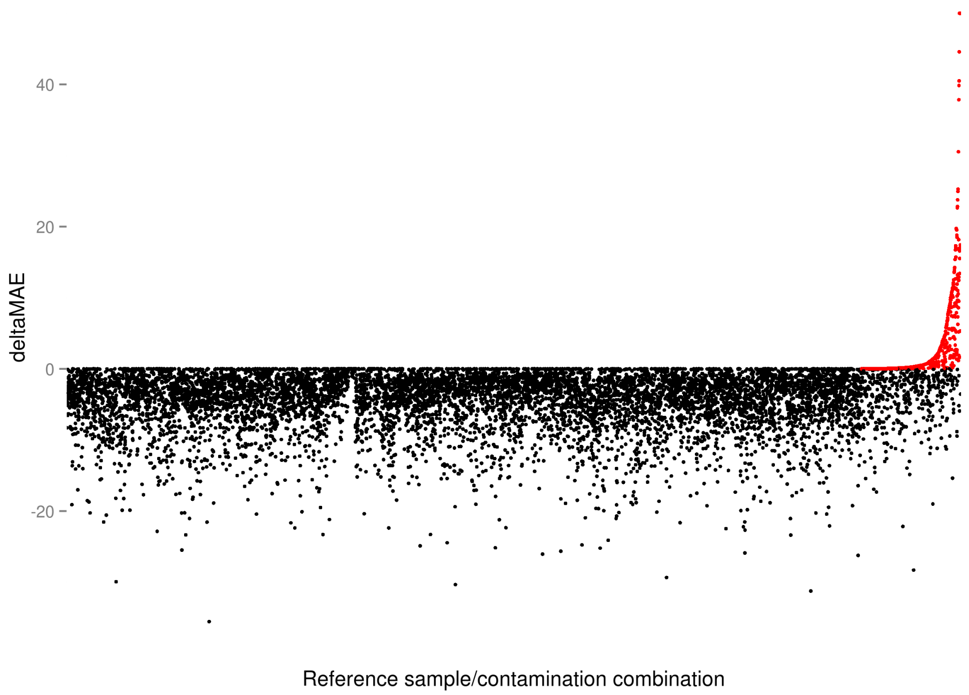

Figure 5: Reference sample dependency of the algorithm. Data from Figure 3, 'median' subset. Red: simulations with worse MAE after correction.

References

1. Yadav VK, De S: **An assessment of computational methods for estimating purity and clonality using genomic data derived from heterogeneous tumor tissue samples.** *Brief Bioinform* 2015, **16:**232-241.
2. GTEx Consortium: **Human genomics. The Genotype-Tissue Expression (GTEx) pilot analysis: multitissue gene regulation in humans.** *Science* 2015, **348:**648-660.
3. Uhlen M, Fagerberg L, Hallstrom BM, Lindskog C, Oksvold P, Mardinoglu A, Sivertsson A, Kampf C, Sjostedt E, Asplund A, et al: **Proteomics. Tissue-based map of the human proteome.** *Science* 2015, **347:**1260419.
4. Newman AM, Liu CL, Green MR, Gentles AJ, Feng W, Xu Y, Hoang CD, Diehn M, Alizadeh AA: **Robust enumeration of cell subsets from tissue expression profiles.** *Nat Methods* 2015, **12:**453-457.
5. Gong T, Szustakowski JD: **DeconRNASeq: a statistical framework for econvolution of heterogeneous tissue samples based on mRNA-Seq data.** *Bioinformatics* 2013, **29:**1083-1085.
6. Shen Q, Hu J, Jiang N, Hu X, Luo Z, Zhang H: **contamDE: Differential expression analysis of RNA-seq data for contaminated tumor samples.** *Bioinformatics* 2015.
7. Wang N, Gong T, Clarke R, Chen L, Shih Ie M, Zhang Z, Levine DA, Xuan J, Wang Y: **UNDO: a Bioconductor R package for unsupervised deconvolution of mixed gene expressions in tumor samples.** *Bioinformatics* 2015, **31:**137-139.
8. Li Y, Xie X: **A mixture model for expression deconvolution from RNA-seq in heterogeneous tissues.** *BMC Bioinformatics* 2013, **14 Suppl 5:**S11.
9. Yoshihara K, Shahmoradgoli M, Martinez E, Vegesna R, Kim H, Torres-Garcia W, Trevino V, Shen H, Laird PW, Levine DA, et al: **Inferring tumour purity and stromal and immune cell admixture from expression data.** *Nat Commun* 2013, **4:**2612.
10. Quon G, Haider S, Deshwar AG, Cui A, Boutros PC, Morris Q: **Computational purification of individual tumor gene expression profiles leads to significant improvements in prognostic prediction.** *Genome Med* 2013, **5:**29.
11. Ahn J, Yuan Y, Parmigiani G, Suraokar MB, Diao L, Wistuba, II, Wang W: **DeMix: deconvolution for mixed cancer transcriptomes using raw measured data.** *Bioinformatics* 2013, **29:**1865-1871.
12. Erkkila T, Lehmusvaara S, Ruusuvuori P, Visakorpi T, Shmulevich I, Lahdesmaki H: **Probabilistic analysis of gene expression measurements from heterogeneous tissues.** *Bioinformatics* 2010, **26:**2571-2577.
13. Kuhn A, Thu D, Waldvogel HJ, Faull RL, Luthi-Carter R: **Population-specific expression analysis (PSEA) reveals molecular changes in diseased brain.** *Nat Methods* 2011, **8:**945-947.
