# Supplementary material for: A transcriptome-based global map of signaling pathways in the ovarian cancer microenvironment associated with clinical outcome
Source: Genome Biol. 2016 May 23;17:108. doi: 10.1186/s13059-016-0956-6 (PMC4877997; doi:10.1186/s13059-016-0956-6)
Supplement: Additional file 2: — Supplementary Figures S1–S4. (PDF 946 kb) [file 13059_2016_956_MOESM2_ESM.pdf]

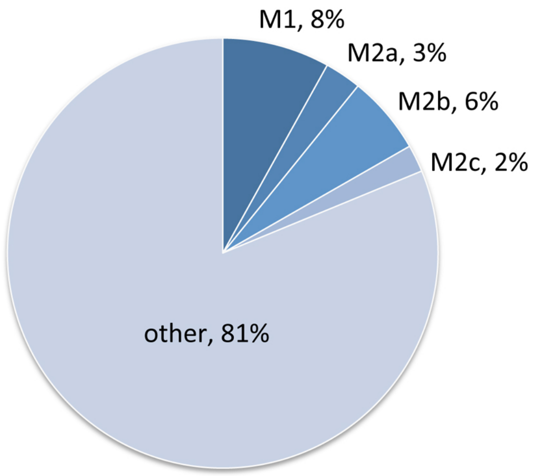


**Figure S1**

Overlap of genes upregulated in ovarian cancer TAMs relative to non-polarized MDMs (M0) (Schumann *et al.,* 2015 and present study) with genes upregulated in MDMs polarized with LPS+INFγ (M1), IL-13 (M2a), ultrapure LPS + immune complexes (M2b) or IL-10 (M2c) (Xue *et al.,* 2014).

A total of 1710 genes were upregulated (fold change >1.5) in TAMs versus non-polarized MDMs (median TPM>3 in TAMs).

Analysis of the MDM data sets published by Xue *et al.* (2014) yielded the following groups (minimum fold change 1.5):
M1, n=871; M2a, n=464; M2b, n=428; M2c, n= 179.

The overlaps of these groups with genes upregulated in TAMs were as follows:
M1, n=138 (8.1%); M2a, n=47 (2.7%); M2b, n=101 (5.9%); M2c, n= 35 (2.0%).
No overlap ("other") was detectable for the majority of genes upregulated in TAMs (81%).

**References**

Schumann T, Adhikary T, Wortmann A, Finkernagel F, Lieber S, Schnitzer E, Legrand N, Schober Y, Nockher WA, Toth PM, et al: **Deregulation of PPARβ/δ target genes in tumor-associated macrophages by fatty acid ligands in the ovarian cancer microenvironment.** *Oncotarget* 2015, **6:**13416-13433.

Xue J, Schmidt SV, Sander J, Draffehn A, Krebs W, Quester I, De Nardo D, Gohel TD, Emde M, Schmidleithner L, et al: **Transcriptome-based network analysis reveals a spectrum model of human macrophage activation.** *Immunity* 2014, **40:**274-288.

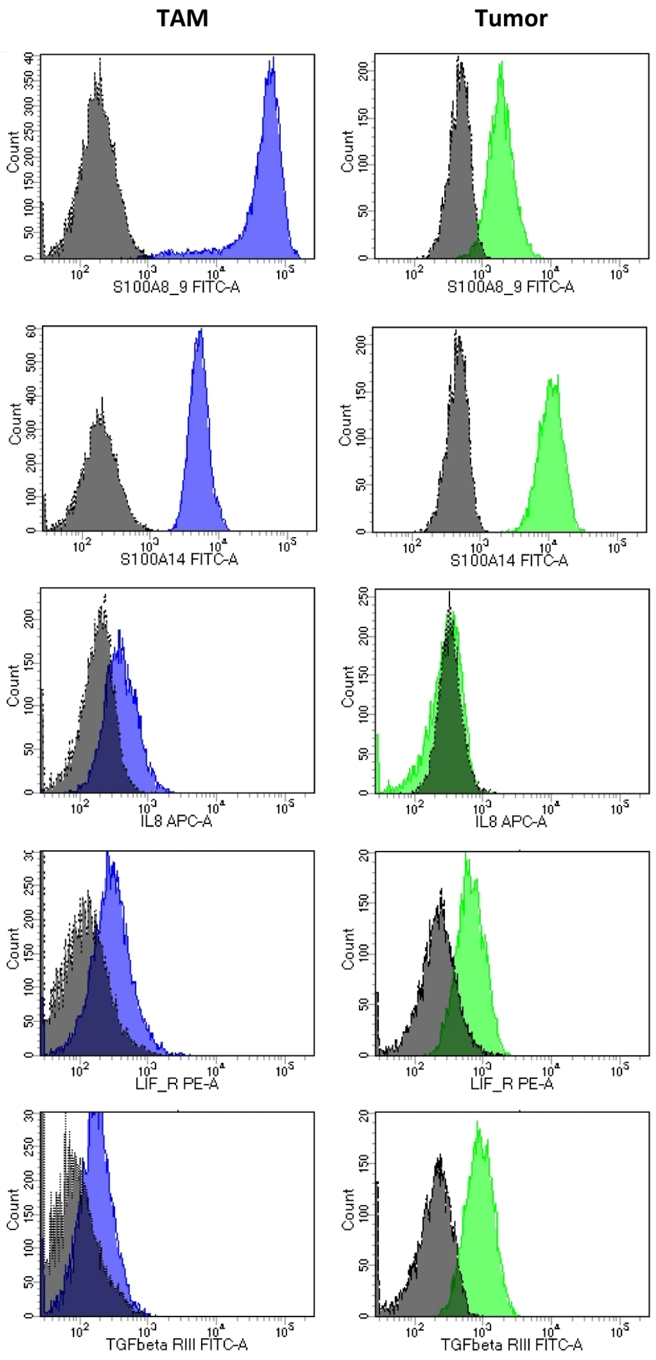


**Figure S2**

FACS histograms of intracellular cytokine expression (S100A8/A9, S100A14, IL-8) and surface receptor exposure (LIFR, TGFBR3) by TAMs (blue peaks) and tumor cells (green).

Grey areas show the staining with matched isotype control antibodies.

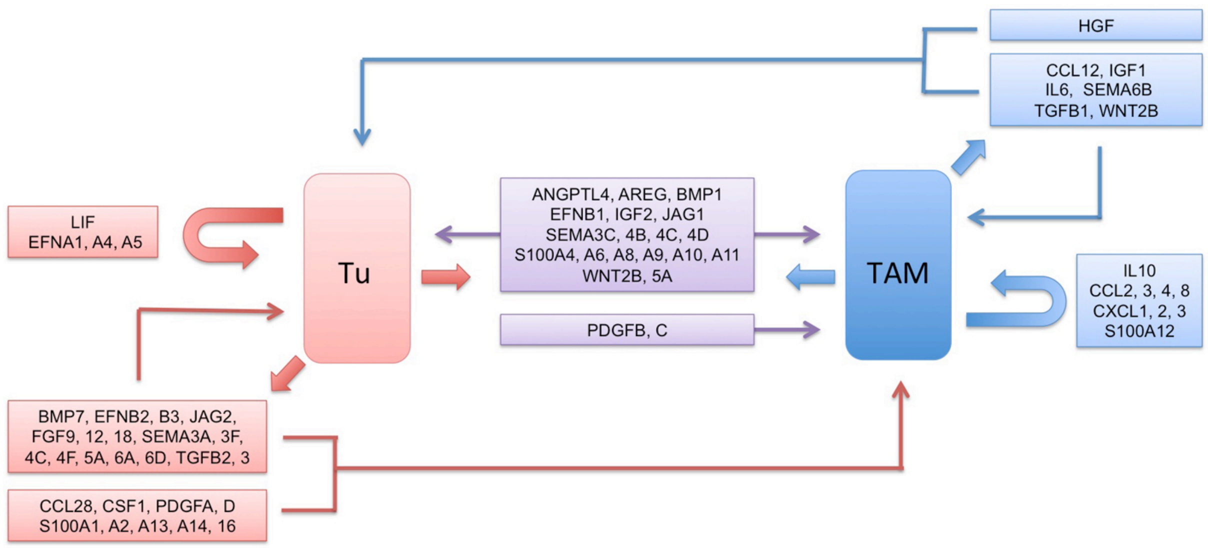


**Figure S3**

Cellular origins and targets of cytokines and growth factors in the ovarian cancer microenviroment.

The model is based on the RNA-Seq data in Figure 2 and the scheme in Figure 4. Gene names are explained in Dataset S1.

Red: ligands mainly expressed by tumor cells; blue: ligands mainly expressed by TAMs; purple: ligands expressed by both cell types.

Thin arrows point to the direction of the major target cell; straight broad arrows indicate the origin of the ligands; bend arrows show autocrine mechanisms.

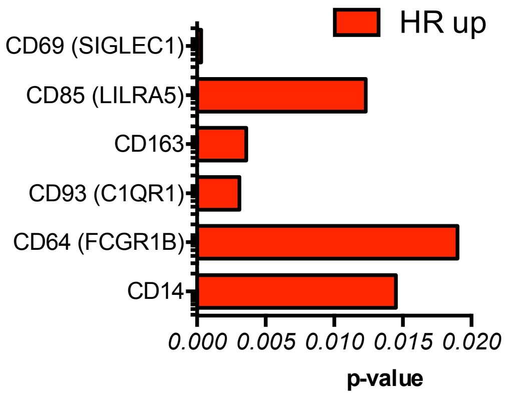


**Figure S4**

Association of relapse-free survival (RFS) with the expression of macrophage genes.

Summary of RFS analyses for 1018 serous ovarian carcinoma patients depicting the results of logrank P tests and the directions of the respective hazard ratio (HR), using the KM plotter database and online tool [37]. Significant instances (for 'JetSet best probe') are shown in red (HR>1) or green (HR<1); grey bars indicate lack of significant associations (p≥0.5) or p > Benjamini-Hochberg critical value for FDR=0.10.
