# Supplementary material for: A transcriptome-based global map of signaling pathways in the ovarian cancer microenvironment associated with clinical outcome
Source: Genome Biol. 2016 May 23;17:108. doi: 10.1186/s13059-016-0956-6 (PMC4877997; doi:10.1186/s13059-016-0956-6)
Supplement: Additional file 6: — Assembly of gene sets. (PDF 200 kb) [file 13059_2016_956_MOESM6_ESM.pdf]

Assembly of cytokine gene set

Ensembl revision 74 genes were filtered for non-protein coding genes. Selected genes were the union of the following sets A, B, C and D, minus a set of manually excluded genes (set E).

Set A was selected by name and description:

the name had to contain "*ADIPOQ, AMH, BMP, DLL, FST, GDF, GHRL, LIF, RSPO, TNF"*,

or the description had to contain "*angiop, chemokine, determination, ephrin-, growth factor, hedgehog, hormone, inhibin, interferon, interleukin, notch, regulin, S100, secreted, wingless"*

or the name had to contain "*LG"* and the description "*ligand"*.

Genes containing "*antisense, carrier, induc, kinase, lipase, nuclear, readthrough, receptor, regulat, respons, stimulat, transcription, zinc*" in their description were filtered, as were genes with an empty ("--") description.

Set B was selected by GO annotation (also retrieved from Ensembl 74). Genes were kept if they were annotated as one of (*chemokine activity, cytokine activity, growth factor activity, hormone activity*) or if they were annotated as *extracellular region* and one of (*BMP signaling pathway, chemoattractant activity, laminin-1 complex, receptor agonist activity, SMAD protein signal transduction, tumor necrosis factor-mediated signaling pathway, type I interferon signaling pathway, vascular endothelial growth factor signaling pathway, Wnt signaling pathway*).

Set C was selected via the annotation to *BIOCARTA_CYTOKINE_PATHWAY* in the Molecular signature database (Subramanian *et al.*, 2005, Proc Natl Acad Sci U S A *102*: 15545-15550).

Set D was build manually and contained the following genes: *SEMA4A, SEMA4B, SEMA4C, SEMA4D, SEMA4F, SEMA4G, SEMA5A, SEMA5B, SEMA6A, SEMA6B, SEMA6C, SEMA6D, SEMA7A, SLIT1, SLIT2,* and *SLIT3.*

Set E (manually removed): *ADCYAP1, AGT, AIMP1, AL512503.1, AMBN, AMELX, AP001888.1, APITD1, APITD1-CORT, C19orf80, C5, CAMLG, CCL15-CCL14, CD320, CECR1, CLEC11A, COPA, CRHR2, CRLF1, CTB-60B18.6, CYFIP2, ENDOU, ESM1, F2, FIBP, FSCN2, FSCN2, GDF5OS, GPI, GUCA2A, HAMP, HBEGF, HMGB1, IAPP, ILF2, ILF3, INS-IGF2, KL, LRSAM1, LTBP1, LTBP2, LTBP3, LTBP4, MACC1, MREG, NAMPT, NOTCH1, NOTCH2, NOTCH2NL, NOTCH3, NOTCH4, NUDT6, OSGIN1, RABEP1, RABEP2, SAMHD1, SBNO1, SBNO2, SHBG, TFF1, TNFSF12-TNFSF13, TTR, TXLNA, TYMP, VSTM1,* and *WISP3.*

Assembly of cytokine receptor gene set

Ensembl revision 74 genes were filtered for non-protein coding genes. Cytokines were matched to receptors by a multi stage process. First, each cytokine gene name was extended with an "R", and resulting existing genes were assigned. Then KEGG (reference) pathway hsa04060 was used to map further cytokine / cytokine receptor pairs. WNT co-receptors were associated by filtering to GO annotations *G-protein coupled receptor activity* and containing *Wnt*. Further genes from the GO annotation groups (*BMP signaling pathway, frizzled binding, transforming growth factor beta receptor signaling pathway, tumor necrosis factor-mediated signaling pathway, Wnt-activated receptor activity*) were assigned, if they

were also annotated as either *plasma membrane* or *cell surface* and their description contained the word *receptor*. Further genes were assigned by manual literature search: *ACVR1, ACVR1B, ACVR2B, ACVRL1, ACVRL1C, ADIPOR1, ADIPOR2, ALK, AMHR2, BMPR1A, BMPR1B, C1QBP, CALCRL, CCKAR, CCKBR, CCR4, CCR5, CCR8, CD127, CD29, CD4, CD7, CD74, CDH13, CFC1, CHL1, CHRNA7, CNTF, CRHR1, CRHR2, CRLF2, CSF1R, CSF2RA, CSF2RB, CXCR2, CXCR3, CXCR4, EDNRA, EDNRB, EGFR, EPHA1, EPHA10, EPHB1, EPHA2, EPHA3, EPHA4, EPHA5, EPHA6, EPHA7, EPHA8, EPHA9, EPHB2, EPHB3, EPHB4, EPHB5, EPHB6, ERBB2, ERBB3, ERBB4, EXTL3, FBLN1), FGFR1, FGFR2, FGFR3, FGFR4, FLT4, FPR1, FSHR, GALR1, GALR2, GALR3, GFRA1, GFRA2, GFRA3, GFRA4, GHSR, GNRHR, GPR30, GPR35, IFNAR1, IFNAR2, IFNGR1, IFNLR1, IGF1, IGF2, IL10R2, IL10RA, IL10RB, IL11RA, IL12RB1, IL13RA1, IL13RA2, IL15RA, IL17RA, IL17RB, IL17RC, IL17RD, IL17RE, IL18R1, IL18RAP, IL1R1, IL1R2, IL1RAP, IL1RL1, IL1RL2, IL20RA, IL23R, IL27RA, IL2RA, IL2RB, IL2RG, IL31RA, IL3RA, IL5RA, IL6ST, INSRR, ITGA4, ITGAM, ITGB3, ITPR1, KREMEN1, L1CAM, LHCGR, LIFR, LRP1, LRP2, MC1R, MC4R, MCHR1, MCRHR2, NGFR, NOTCH1, NOTCH2, NPR1, NPR2, NPR3, NPY1R, NPY2R, NPY4R, NPY5R, NRCAM, NRP1, NRP2, NTN1, NTN3, NTRK1, NTRK2, PDGFRB, PITPNM3, PLXNA1, PLXNA2, PLXNA3, PLXNA4, PLXNB1, PLXND1, PPYR1, PROKR1, PTCH1, PTCH2, PTGFR, PTH1R, PTPRZ1, RAMP2, RAMP3, ROBO1, RXFP1, RXFP2, RXFP3, RXFP4, SDC1, SDC3, SDC4, SELP, SIGIRR, SSTR1, SSTR2, SSTR3, SSTR4, SSTR5, SSTR6, TGFBR1, TLR4, TLR9, TNFRSF1A, TRHR, TRKB, TRKC, TSHR, UTS2R, VIPR1, VIPR2.*

Assembly of lipid gene set

Ensembl revision 74 genes were filtered for non-protein coding genes. Genes with "*acetyl-CoA, acyltransferase, antisense, cardiolipin, glycine, hedgehog, malonyl, patatin, phosphatase, pseudogene, readthrough, receptor, scramblase, sterol, transfer, transporter, wax*" in their description were removed. Only genes with descriptions matching "*acyltransferase, leukotriene, lipase, lipoxygenase, lysophosphatidic, phospholipase A2, prostaglandin*" or name matching "*CYP2C8, CYP2C9, CYP2E1, CYP2J2, CYP4A11, CYP4F2, CYP4F3, ENPP2, EPHX2, GPX4 , GPX4, LPPR, MGS2 , MGST3 , PLAA, THAS*" were kept.

Assembly of lipid receptor gene set

Ensembl revision 74 genes were filtered for non-protein coding genes with "*receptor"* in their description. Genes with "*acyltransferase, antisense, coactivator, pseudogene, readthrough, transfer*" in their description were removed. Only genes with descriptions matching "*eicosanoid, leukotriene, lipoxygenase, lysophos, phospholip, prostaglandin"* or name matching "*PPARA, PPARD, PPARG"* were kept.
